# Genetically predicted high sex hormone binding globulin was associated with decreased risk of polycystic ovary syndrome

**DOI:** 10.1186/s12905-024-03144-6

**Published:** 2024-06-20

**Authors:** Xiaofeng Guo, Langlang Chen, Jianhua He, Xiaozhi Zhang, Shui Xu

**Affiliations:** The Second Affiliated Hospital of Zhejiang University School of Medicine, LanXi Hospital; LanXi People’s Hospital, Jinhua, 321100 China

**Keywords:** Mendelian randomization, Polycystic ovary syndrome, Sex hormone-binding globulin, Single nucleotide polymorphism

## Abstract

**Background:**

Previous observational studies have indicated an inverse correlation between circulating sex hormone binding globulin (SHBG) levels and the incidence of polycystic ovary syndrome (PCOS). Nevertheless, conventional observational studies may be susceptible to bias. Consequently, we conducted a two-sample Mendelian randomization (MR) investigation to delve deeper into the connection between SHBG levels and the risk of PCOS.

**Methods:**

We employed single-nucleotide polymorphisms (SNPs) linked to serum SHBG levels as instrumental variables (IVs). Genetic associations with PCOS were derived from a meta-analysis of GWAS data. Our primary analytical approach relied on the inverse-variance weighted (IVW) method, complemented by alternative MR techniques, including simple-median, weighted-median, MR-Egger regression, and MR Pleiotropy RESidual Sum and Outlier (MR-PRESSO) testing. Additionally, sensitivity analyses were conducted to assess the robustness of the association.

**Results:**

We utilized 289 SNPs associated with serum SHBG levels, achieving genome-wide significance, as instrumental variables (IVs). Our MR analyses revealed that genetically predicted elevated circulating SHBG concentrations were linked to a reduced risk of PCOS (odds ratio (OR) = 0.56, 95% confidence interval (CI): 0.39–0.78, *P* = 8.30 × 10^–4^) using the IVW method. MR-Egger regression did not detect any directional pleiotropic effects (*P* intercept = 0.626). Sensitivity analyses, employing alternative MR methods and IV sets, consistently reaffirmed our results, underscoring the robustness of our findings.

**Conclusions:**

Through a genetic epidemiological approach, we have substantiated prior observational literature, indicating a potential causal inverse relationship between serum SHBG concentrations and PCOS risk. Nevertheless, further research is needed to elucidate the underlying mechanism of SHBG in the development of PCOS.

**Supplementary Information:**

The online version contains supplementary material available at 10.1186/s12905-024-03144-6.

## Background

Polycystic ovary syndrome (PCOS) is the most common endocrinopathy in women of childbearing age, characterized by hyperandrogenism, menstrual disorders, and polycystic ovarian changes [1, 2]. It was estimated that in 2019, approximately 66.0 million individuals were affected by PCOS worldwide and the overall prevalence rate was 829.6 per 100,000 [3]. Patients with PCOS may not only have a higher rate of infertility but also have an increased risk of developing diabetes and cardiovascular diseases, which poses a great challenge to public health management [2].

While the exact cause and development of PCOS remain unclear, evidence suggests that a combination of genetic and environmental factors significantly contribute to its etiology and pathogenesis. Established risk factors for PCOS encompass genetic susceptibility, obesity, and insulin resistance [4–7]. Sex hormone binding globulin (SHBG) acts as a carrier for sex hormones, binding with both testosterone and estrogen, and holds a crucial role in various physiological and pathological contexts [8]. Observational studies have consistently shown lower circulating SHBG levels in PCOS patients when compared to healthy controls. For instance, in a study involving 200 PCOS patients and 200 controls, peripheral SHBG levels were significantly higher in the control group than in the PCOS group [9]. Another study, which included 585 women with PCOS and 171 controls, similarly observed lower serum SHBG levels in PCOS patients compared to controls [10]. However, since findings from traditional observational epidemiological studies are susceptible to bias such as confounding and reverse causation, it remains unclear whether the observed association was causal or not.

Mendelian randomization (MR) is a genetic epidemiological approach that utilizes single nucleotide polymorphisms (SNPs) strongly linked to the exposure as instrumental variables (IVs) to assess potential causal relationships between the exposure and the outcome [11]. Since genotypes are assumed to be randomly distributed during gamete formation, the application of instrumental variable models effectively addresses the issue of confounding in observational studies, particularly the potential bias caused by unmeasured confounding variables in causal inference [12, 13]. Furthermore, as genotypes precede the onset of disease, MR studies can effectively mitigate the issue of reverse causation. Consequently, in this study, we conducted a two-sample MR investigation to explore the potential link between circulating SHBG levels and the risk of PCOS.

## Methods

### Selection of genetic variants

**Fig. 1:**
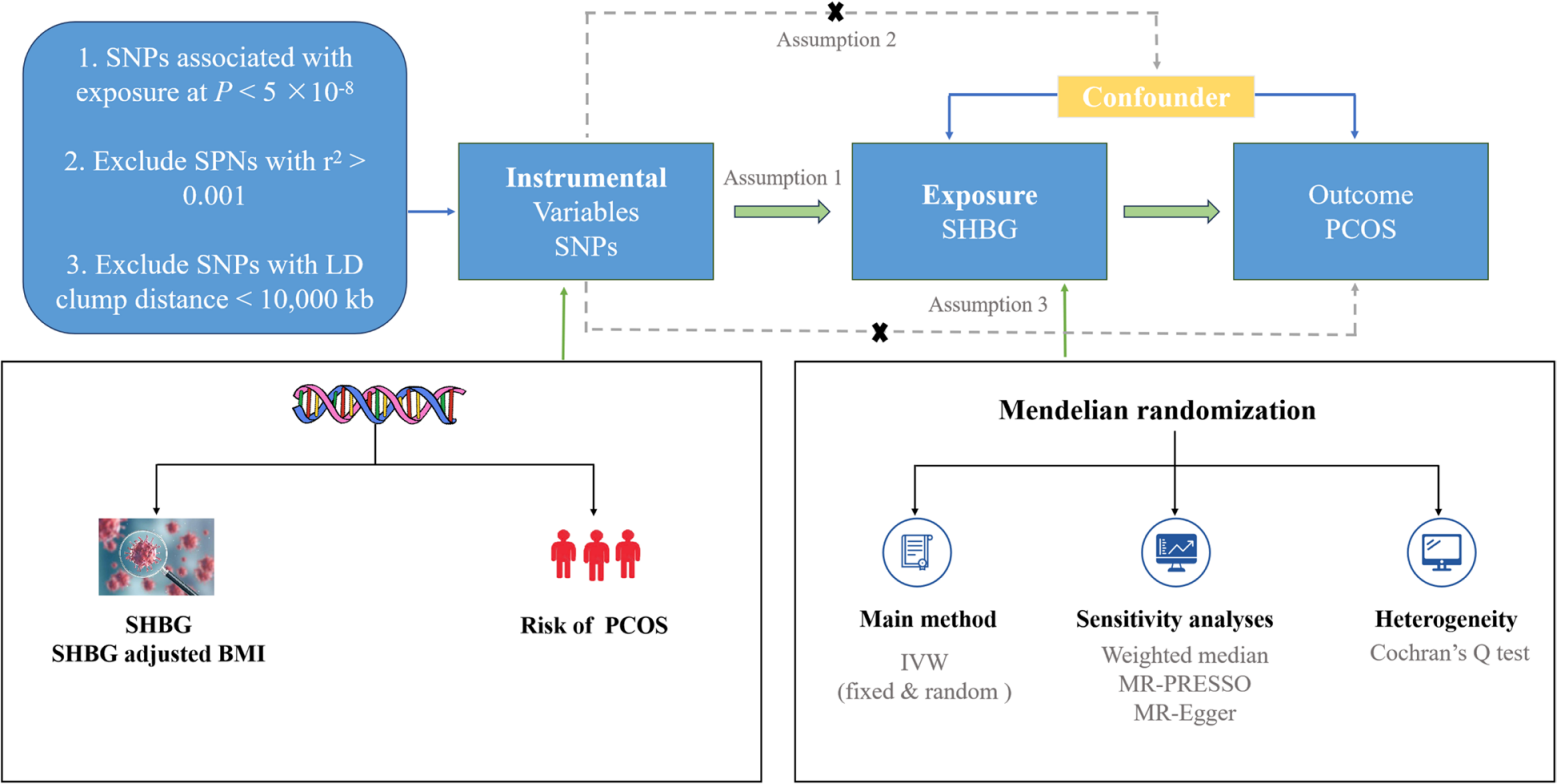
An overall design of the present study. Abbreviations: IVW, inverse-variance weighted; GWAS, Genome-wide association study; MR, Mendelian randomization; MR-PRESSO, MR pleiotropy residual sum and outlier test; PCOS, polycystic ovary syndrome; SHBG, sex hormone binding globulin; SNP, single nucleotide polymorphism

The overall design of this study is shown in Fig. 1. The IVs for serum SHBG were obtained from a recent genome-wide association study (GWAS) involving 425,097 individuals of European descent [14]. A total of 305 independent SNPs (r^2^ threshold < 0.001, kb = 10,000) associated with circulating SHBG levels at genome-wide significance level (*P* < 5 × 10^−8^) were identified. Of these, 16 were not present in the primary outcome dataset. Consequently, we ultimately utilized 289 SNPs associated with circulating SHBG concentrations as instrumental variables (IVs) in the subsequent MR analyses. The details of GWAS studies and datasets used in the present study are listed in Supplementary Table 1, and the detailed information of the selected SNPs used as IVs is displayed in Supplementary Table 2.

Furthermore, as body mass index (BMI) has been shown to be correlated with both circulating SHBG levels and PCOS risk, we selected 462 independent SHBG-associated SNPs (with an r^2^ threshold < 0.001 and kb = 10,000) from the same GWAS meta-analysis, after adjusting for BMI (*P* < 5 × 10^−8^). Among them, 22 SNPs were not available in the outcome dataset. Finally, 440 SNPs associated with circulating SHBG concentrations adjusted by BMI were used as IVs in the primary analyses. The detailed information of the selected SNPs used as IVs is displayed in Supplementary Table 3.

### Outcome dataset

The genetic association data for PCOS were sourced from a GWAS meta-analysis, which encompassed 10,074 cases and 103,164 controls of European ancestry [15]. This represents the most extensive GWAS meta-analysis of PCOS to date, incorporating participants from seven cohorts, including Rotterdam, EGCUT, deCODE, UK (London/Oxford), Chicago, Boston, and 23andMe [15]. However, due to unavailability of data from 23andMe, the final study population for this investigation comprised 4,138 PCOS patients and 20,129 controls [15]. Comprehensive details of these studies have been previously documented in published articles, and all participating studies received approval from their respective ethical committees. Furthermore, we selected another summary data associated with PCOS involving 1,424 cases and 200,581 controls from the Finland consortium (https://www.finngen.fi/fi), which collected and analyzed genetic and health related data from about 500,000 participants to replicate our findings.

### Statistical analysis

We first calculated F-statistics to quantify the strength of the IVs, with the equation of F = beta^2^ /se^2^ [16]. A F-statistic greater than 10 suggests the IVs are unlikely to suffer from weak instrument bias.

Our primary analysis employed the inverse-variance weighted (IVW) method to investigate the potential causal association between circulating SHBG concentrations and PCOS risk. This method initially derives causal effect estimates from individual genetic instrumental variables, namely, the effect estimates of each SNP on both SHBG and PCOS. These estimates are then aggregated through meta-analysis to yield a consolidated causal effect estimate [17]. The estimated value from the IVW method is essentially equivalent to the regression coefficient in weighted regression with zero intercept term [18, 19]. In addition, we conducted a series of alternative MR methods to analyze the effect of potential pleiotropy on causal estimation. For instance, MR-Egger regression was used to evaluate influence of potential directional pleiotropy. In MR-Egger regression analysis, the intercept term signifies the average pleiotropic effect of genetic variation [20]. If the intercept is different from zero, there is evidence of directed pleiotropy [20]. Under the assumption that the correlation between genetic variation and exposure is unrelated to the direct impact of genetic variation on the outcomes, the slope coefficient in MR-Egger regression provides a consistent estimate of the causal effect [20]. Furthermore, we employed both the simple-median and weighted-median methods, which involve combining either unweighted or weighted estimates using the median. As long as the weight of the causal effect calculated by the effective instrumental variable reaches 50%, a consistent estimation of the causal effect can be obtained [21]. Moreover, the likelihood-based method was used to evaluate the linear relationship between the exposure and the outcome, and the likelihood-based estimator expresses the causal increase in the outcome per unit change in the risk factor assuming a linear association between the risk factor and the outcome variables [22]. Lastly, we employed the Mendelian randomization pleiotropy residual sum and outlier (MR-PRESSO) method to identify and address horizontal pleiotropic outliers. This method conducts a global heterogeneity test by regressing the SNP-outcome (PCOS) associations against the SNP-exposure (SHBG) associations and then comparing the actual distance of each SNP from the regression line with the expected distance under the null hypothesis of no pleiotropy [23].

All statistical analyses were performed using R (version 3.6.3) with packages “MendelianRandomization” and “MR-PRESSO”, unless otherwise noted. An observed *P*-value < 0.013(0.05/2/2) was considered as statistically significant evidence for a causal association by using Bonferroni correction. A *P*-value ranging from 0.013 to 0.05 was considered as suggestive evidence.

## Results

In the present study, a total of 289 and 440 independent SNPs associated with SHBG and SHBG adjusted BMI achieving genome-wide significance were used as IVs in the primary analysis. The *F-statistic* for SHBG and SHBG adjusted BMI was 21.77–1857.13, satisfying the threshold of > 10.

**Fig. 2:**
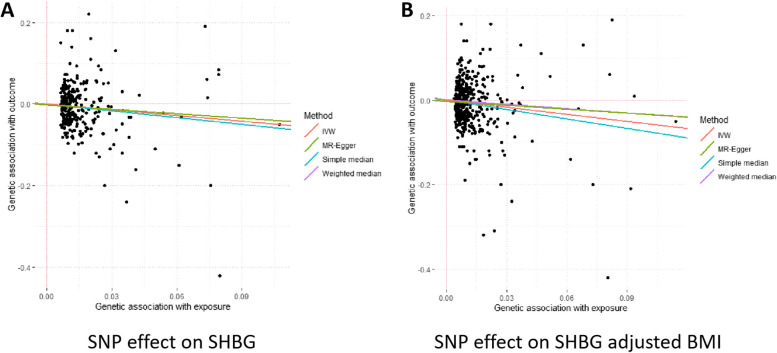
Association between genetically predicted SHBG levels, SHBG adjusted BMI levels and risk of PCOS based on different MR methods. Abbreviations: BMI, Body mass index; IVW, inverse-variance weighted; MR, Mendelian randomization; SHBG, sex hormone binding globulin; SNP, single nucleotide polymorphism

As shown in Table 1, genetically predicted higher levels of circulating SHBG were significant associated with a decreased risk of PCOS. For one standard deviation (SD) (approximately 30.3 nmol/L) increment of SHBG levels, the odds ratio (OR) of PCOS was 0.56 [95% confidence interval (CI): 0.39–0.78, *P* = 8.30 × 10^–4^]. The causal effect estimate was consistent in the sensitivity analyses using the simple-median (OR = 0.51, 95% CI: 0.30–0.87,* P* = 0.013), weighted-median (OR = 0.60, 95% CI: 0.35–1.03,* P* = 0.066) and Maximum-likelihood (OR = 0.55, 95% CI: 0.39–0.78,* P* = 7.28 × 10^–4^) methods. One outlier SNPs were detected using MR-PRESSO test, and the causal effect estimate between SHBG and PCOS was similar (OR = 0.54, 95% CI: 0.39–0.76, *P* = 4.07 × 10^–4^). MR-Egger regression did not suggest evidence of potential directional pleiotropy (*P* for intercept = 0.626) (Fig. 2). Since BMI has been reported to be associated with both risk of PCOS and levels of circulating SHBG, we repeated our MR analysis using SHBG associated SNPs with adjustments for BMI as IVs to further evaluate the robustness of our main analyses. The causal effect of circulating SHBG adjusted for BMI with risk of PCOS remained consistent (OR = 0.53, 95% CI: 0.38–0.73, *P* = 4.07 × 10^–4^, by IVW method) (Fig. 2).

**Table 1:** Genetically predicted circulating SHBG levels and risk of PCOS

Traits/Methods	No. of SNPs	OR (95% CI)	*P* for association	*P* for heterogeneity	*P* intercept from MR-Egger regression
SHBG					
Inverse-variance weighted	289	0.56 (0.39–0.78)	8.30 × 10^–4^	0.006	
Simple-median	289	0.51 (0.30–0.87)	0.013		
Weighted-median	289	0.60 (0.35–1.03)	0.066		
Maximum-likelihood	289	0.55 (0.39–0.78)	7.28 × 10^–4^		
MR-PRESSO test	288	0.56 (0.39–0.78)	9.40 × 10^–4^		
MR-Egger	289	0.48 (0.25–0.94)	0.031		0.626
SHBG adjusted BMI					
Inverse-variance weighted	440	0.53 (0.38–0.73)	9.84 × 10^–5^	0.057	
Simple-median	440	0.42 (0.24–0.73)	0.003		
Weighted-median	440	0.69 (0.40–1.22)	0.203		
Maximum-likelihood	440	0.53 (0.38–0.75)	2.57 × 10^–4^		
MR-PRESSO test	440	0.53 (0.38–0.71)	2.44 × 10^–4^		
MR-Egger	440	0.53 (0.38–0.74)	0.278		0.212

*Abbreviations: CI* Confidence interval, *No* Number, *MR* Mendelian randomization, *MR-PRESSO test* MR-Pleiotropy RESidual Sum and Outlier test, *OR* Odds ratio, *SHBG* Sex hormone binding globulin, *PCOS* Polycystic ovary syndrome, *SNP* Single nucleotide polymorphis

When replicating the findings in the Finnegan consortium dataset, we observed consistent results for the association between SHBG and SHBG-adjusted BMI using the IVW method (OR = 0.45, 95% CI: 0.30–0.68, *P* = 1.83 × 10^–4^ for SHBG and OR = 0.50, 95% CI: 0.33–0.75, *P* = 8.87 × 10^–4^ for SHBG-adjusted BMI). The results of the other sensitivity analyses are listed in Supplementary Table 4.

## Discussion

In this study, a two-sample MR approach was employed to assess the potential causal link between circulating SHBG levels and PCOS risk. The analysis revealed that genetically predicted high SHBG levels were linked to a reduced PCOS risk. Our findings remained consistent across various sensitivity analyses using different MR methods and alternative instrumental variable sets, underscoring their robustness.

SHBG, a serum protein linked to sex hormones, plays a critical role in facilitating the action and transport of these hormones [24]. Its levels have been documented to correlate with PCOS development, as well as with complications and long-term outcomes in PCOS patients. A reduced serum SHBG level in PCOS patients serves as a significant risk factor for hyperandrogenemia and serves as a crucial predictor of insulin resistance, as well as disturbances in glucose and lipid metabolism [25]. Observational epidemiological studies have provided evidence of an association between SHBG and PCOS risk. Cross-sectional studies have further indicated that SHBG levels are lower in the PCOS group compared to the control group [26, 27]. In a case–control study, the mean SHBG concentration was notably lower in the PCOS group compared to the control group, with statistically significant differences observed (*P* = 0.004) [28]. In addition, in a recent meta-analysis comprising 5,121 cases and 5,059 controls, it was reported that circulating SHBG levels were lower in PCOS patients compared to controls (SMD = -0.83, 95%CI = -1.01 to -0.64) [29]. Consistently, our findings support the notion of a protective effect of circulating SHBG levels against the development of PCOS.

While the precise molecular mechanism by which SHBG contributes to PCOS development remains unclear, various studies have indicated potential biological pathways involved in the pathogenesis of PCOS. For instance, the reduction in SHBG levels among PCOS patients results in elevated free and bioactive androgen levels, increased luteinizing hormone secretion, and reduced follicle-stimulating hormone secretion. These factors collectively lead to a high rate of follicle atresia and ultimately contribute to ovulation disorders [30, 31]. Moreover, insulin resistance (IR) stands out as a prominent characteristic of PCOS [32]. In a study by Fu Chen et al., SHBG was identified as an independent influential factor for HOMA-IR and could serve as a valuable predictive marker for IR in PCOS patients [33]. Feng C et al. conducted in vitro studies using a human insulin-resistant cell model, demonstrating that SHBG may down-regulate the Phosphatidylinositol 3 kinase/protein kinase B (PI3K/AKT) pathway. This pathway is known to be associated with the development of both local and systemic insulin resistance, which is considered to play a role in the connection between metabolic disorders and reproductive dysfunction in PCOS [34]. While these explanations hold biological plausibility, further studies are needed to clarify the precise role of SHBG in PCOS development.

Prior investigations have also indicated an association between BMI and both SHBG levels and PCOS risk. For instance, in a population-based cohort study, it was observed that women who were overweight or obese at both 14 and 31 years of age had a heightened risk of PCOS development [relative risk (RR) = 1.71, 95% CI = 1.30–2.24] [35]. Another study reported that SHBG correlated negatively with BMI in both PCOS (*P* < 0.0001) and non-PCOS groups (*P* = 0.001) [36]. Hence, in this study, we conducted a sensitivity analysis by employing IVs adjusted for BMI and reran the MR analysis. The causal association between SHBG and PCOS risk remained consistent, bolstering confidence in the potential causal role of SHBG in PCOS development.

There are some strengths in our study when compared with previous studies [14, 37]. To ensure three fundamental assumptions, we selected SNPs from independent loci related to circulating SHBG levels with more rigorous criterion (*P* < 5 × 10^−8^, r^2^ threshold < 0.001, kb = 10,000) from the largest GWAS to date as our IVs [13]. Second, MR analyses employing genetic variants as IVs largely address the issue of confounding inherent in traditional observational studies because genotypes are randomly allocated during gamete formation. We also performed different MR methods to test for potential pleiotropy. We did not observe evidence of directional pleiotropy for the causal association between SHBG and risk of PCOS in any of the above MR approaches. Lastly, we replicated our findings using another set of summary data, yielding consistent results. Nonetheless, this study also encounters limitations. While the majority of participants in the GWAS meta-analysis were of European descent, it's important to acknowledge the potential confounding effects arising from population stratification. Consequently, the results of this study may not be entirely applicable to individuals of non-European descent. Furthermore, we could not assess the potential nonlinear effects of serum SHBG on the risk PCOS by using the MR method. While we try our best to avoid pleiotropy, we cannot totally rule out the possibility of pleiotropy. Canalization plays a crucial role in complex organisms and should be considered when investigating genetic causality through two-sample MR analysis. However, the association between SHBG and the risk of PCOS needs further validation through additional studies due to the absence of individual data.

## Conclusions

In conclusion, our study revealed a genetic association between high SHBG levels and reduced PCOS risk, implying a potential causal role for SHBG in PCOS development. Further investigations are needed to elucidate the underlying mechanisms by which SHBG influences the development of PCOS.

### Supplementary Information

Supplementary Material 1.

## Data Availability

Datasets used for the analysis are available under reasonable requests. Data on SHBG were contributed by Katherine S Ruth et al., (2020) and were downloaded from https://www.nature.com/articles/s41591-020-0751-5#Sec19. Data on PCOS was contributed by Felix Day et al., (2018) and were downloaded from https://doi.org/10.17863/CAM.27720.

## Abbreviations

BMI: Body mass index
CI: Confidence interval
IR: Insulin resistance
IV: Instrumental variables
IVW: Inverse-variance weighted
GWAS: Genome-wide association study
MR: Mendelian randomization
MR-PRESSO: Mendelian randomization pleiotropy residual sum and outlier
OR: Odds ratio
PCOS: Polycystic ovary syndrome
SD: Standard deviation
SHBG: Sex hormone binding globulin
SNP: Single nucleotide polymorphisms
UI: Uncertainty interval

## References

[1] Meier RK (2018). Polycystic Ovary Syndrome. Nurs Clin North Am.

[2] Louwers YV, Laven JSE (2020). Characteristics of polycystic ovary syndrome throughout life. Ther Adv Reprod Health.

[3] Diseases GBD, Injuries C (2020). Global burden of 369 diseases and injuries in 204 countries and territories, 1990–2019: a systematic analysis for the global burden of disease study 2019. Lancet.

[4] Vink JM, Sadrzadeh S, Lambalk CB, Boomsma DI (2006). Heritability of polycystic ovary syndrome in a Dutch twin-family study. J Clin Endocrinol Metab.

[5] Barbieri RL, Sluss PM, Powers RD, McShane PM, Vitonis A, Ginsburg E, Cramer DC (2005). Association of body mass index, age, and cigarette smoking with serum testosterone levels in cycling women undergoing in vitro fertilization. Fertil Steril.

[6] Dunaif A (1997). Insulin resistance and the polycystic ovary syndrome: mechanism and implications for pathogenesis. Endocr Rev.

[7] Traub ML (2011). Assessing and treating insulin resistance in women with polycystic ovarian syndrome. World J Diabetes.

[8] Fortunati N (1999). Sex hormone-binding globulin not only a transport protein What news is around the corner. J Endocrinol Invest..

[9] Bhatnager R, Senwal A, Nanda S, Dang AS (2019). Association of rs6259 polymorphism with SHBG levels and Poly Cystic Ovary Syndrome in Indian population: a case control study. Mol Biol Rep.

[10] Schweighofer N, Lerchbaum E, Trummer O, Schwetz V, Pilz S, Pieber TR, Obermayer-Pietsch B (2012). Androgen levels and metabolic parameters are associated with a genetic variant of F13A1 in women with polycystic ovary syndrome. Gene.

[11] Katan MB (2004). Commentary: mendelian randomization, 18 years on. Int J Epidemiol.

[12] Stukel TA, Fisher ES, Wennberg DE, Alter DA, Gottlieb DJ, Vermeulen MJ (2007). Analysis of observational studies in the presence of treatment selection bias: effects of invasive cardiac management on AMI survival using propensity score and instrumental variable methods. JAMA.

[13] Davies NM, Holmes MV, Davey SG (2018). Reading Mendelian randomisation studies: a guide, glossary, and checklist for clinicians. BMJ.

[14] Ruth KS, Day FR, Tyrrell J, Thompson DJ, Wood AR, Mahajan A, Beaumont RN, Wittemans L, Martin S, Busch AS (2020). Using human genetics to understand the disease impacts of testosterone in men and women. Nat Med.

[15] Day F, Karaderi T, Jones MR, Meun C, He C, Drong A, Kraft P, Lin N, Huang H, Broer L (2018). Large-scale genome-wide meta-analysis of polycystic ovary syndrome suggests shared genetic architecture for different diagnosis criteria. PLoS Genet.

[16] Chong M, Sjaarda J, Pigeyre M, Mohammadi-Shemirani P, Lali R, Shoamanesh A, Gerstein HC, Pare G (2019). Novel drug targets for ischemic stroke identified through mendelian randomization analysis of the blood proteome. Circulation.

[17] Lawlor DA, Harbord RM, Sterne JA, Timpson N, Davey SG (2008). Mendelian randomization: using genes as instruments for making causal inferences in epidemiology. Stat Med.

[18] Burgess S, Butterworth A, Thompson SG (2013). Mendelian randomization analysis with multiple genetic variants using summarized data. Genet Epidemiol.

[19] Thompson SG, Sharp SJ (1999). Explaining heterogeneity in meta-analysis: a comparison of methods. Stat Med.

[20] Bowden J, Davey Smith G, Burgess S (2015). Mendelian randomization with invalid instruments: effect estimation and bias detection through Egger regression. Int J Epidemiol.

[21] Bowden J, Davey Smith G, Haycock PC, Burgess S (2016). Consistent estimation in mendelian randomization with some invalid instruments using a weighted median estimator. Genet Epidemiol.

[22] Burgess S, Scott RA, Timpson NJ, Davey Smith G, Thompson SG, Consortium E-I (2015). Using published data in Mendelian randomization: a blueprint for efficient identification of causal risk factors. Eur J Epidemiol..

[23] Wu F, Huang Y, Hu J, Shao Z (2020). Mendelian randomization study of inflammatory bowel disease and bone mineral density. BMC Med.

[24] Tawfeek MA, Alfadhli EM, Alayoubi AM, El-Beshbishy HA, Habib FA (2017). Sex hormone binding globulin as a valuable biochemical marker in predicting gestational diabetes mellitus. BMC Womens Health.

[25] Yasui T, Tomita J, Miyatani Y, Yamada M, Uemura H, Irahara M, Arai M, Kojimahara N, Okabe R, Ishii Y (2007). Associations of adiponectin with sex hormone-binding globulin levels in aging male and female populations. Clin Chim Acta.

[26] Zhu Q, Zhou H, Zhang A, Gao R, Yang S, Zhao C, Wang Y, Hu J, Goswami R, Gong L (2016). Serum LBP Is Associated with Insulin Resistance in Women with PCOS. PLoS ONE.

[27] Jayagopal V, Kilpatrick ES, Jennings PE, Hepburn DA, Atkin SL (2003). The biological variation of testosterone and sex hormone-binding globulin (SHBG) in polycystic ovarian syndrome: implications for SHBG as a surrogate marker of insulin resistance. J Clin Endocrinol Metab.

[28] Sieminska L, Marek B, Kos-Kudla B, Niedziolka D, Kajdaniuk D, Nowak M, Glogowska-Szelag J (2004). Serum adiponectin in women with polycystic ovarian syndrome and its relation to clinical, metabolic and endocrine parameters. J Endocrinol Invest.

[29] Deswal R, Yadav A, Dang AS (2018). Sex hormone binding globulin - an important biomarker for predicting PCOS risk A systematic review and meta-analysis. Syst Biol Reprod Med..

[30] Cho LW, Jayagopal V, Kilpatrick ES, Holding S, Atkin SL (2006). The LH/FSH ratio has little use in diagnosing polycystic ovarian syndrome. Ann Clin Biochem.

[31] Chang RJ (2007). The reproductive phenotype in polycystic ovary syndrome. Nat Clin Pract Endocrinol Metab.

[32] Cassar S, Misso ML, Hopkins WG, Shaw CS, Teede HJ, Stepto NK (2016). Insulin resistance in polycystic ovary syndrome: a systematic review and meta-analysis of euglycaemic-hyperinsulinaemic clamp studies. Hum Reprod.

[33] Chen F, Liao Y, Chen M, Yin H, Chen G, Huang Q, Chen L, Yang X, Zhang W, Wang P (2021). Evaluation of the Efficacy of Sex Hormone-Binding Globulin in Insulin Resistance Assessment Based on HOMA-IR in Patients with PCOS. Reprod Sci.

[34] Feng C, Jin Z, Chi X, Zhang B, Wang X, Sun L, Fan J, Sun Q, Zhang X (2018). SHBG expression is correlated with PI3K/AKT pathway activity in a cellular model of human insulin resistance. Gynecol Endocrinol..

[35] Laitinen J, Taponen S, Martikainen H, Pouta A, Millwood I, Hartikainen AL, Ruokonen A, Sovio U, McCarthy MI, Franks S (2003). Body size from birth to adulthood as a predictor of self-reported polycystic ovary syndrome symptoms. Int J Obes Relat Metab Disord.

[36] Yasmin E, Balen AH, Barth JH (2013). The association of body mass index and biochemical hyperandrogenaemia in women with and without polycystic ovary syndrome. Eur J Obstet Gynecol Reprod Biol.

[37] Day FR, Hinds DA, Tung JY, Stolk L, Styrkarsdottir U, Saxena R, Bjonnes A, Broer L, Dunger DB, Halldorsson BV (2015). Causal mechanisms and balancing selection inferred from genetic associations with polycystic ovary syndrome. Nat Commun.

