# Supplementary material for: Genetically predicted high sex hormone binding globulin was associated with decreased risk of polycystic ovary syndrome
Source: BMC Womens Health. 2024 Jun 20;24:357. doi: 10.1186/s12905-024-03144-6 (PMC11188236; doi:10.1186/s12905-024-03144-6)
Supplement: Supplementary file 1 — Supplementary Material 1. [file 12905_2024_3144_MOESM1_ESM.docx]

Supplementary Material for

**Genetically predicted high sex hormone binding globulin was associated with decreased risk of polycystic ovary syndrome**

**Supplementary Table 1.** Detailed information of studies and datasets used in the present study.

Abbreviations: PCOS, polycystic ovary syndrome; SHBG, sex hormone binding globulin.

**Supplementary Table 2.** Associations of the genetic variants with circulating SHBG levels and their effect estimates on risk of polycystic ovary syndrome.

Abbreviations: SHBG, sex hormone binding globulin; PCOS, polycystic ovary syndrome; Chr, chromosome; SE, standard error; SNP, single nucleotide polymorphism.

**Supplementary Table 3.** Associations of the genetic variants with SHBG levels adjusted by BMI and their effects on polycystic ovary syndrome.

Abbreviations: SHBG, sex hormone binding globulin; PCOS, polycystic ovary syndrome; BMI, body mass index; Chr, chromosome; SE, standard error; SNP, single nucleotide polymorphism.

**Supplementary Table 4.** Effect estimates of the associations between circulating levels of SHBG, SHBG adjusted BMI and risk of polycystic ovary syndrome in replication study.

Abbreviations: CI, confidence interval; No, Number ; MR, Mendelian randomization; MR-PRESSO test, MR-Pleiotropy RESidual Sum and Outlier test; OR, odds ratio; SHBG, sex hormone binding globulin; PCOS, polycystic ovary syndrome; SNP, single nucleotide polymorphis

**Supplementary Table 1. Detailed information of studies and datasets used in the present study.**

| **Exposure or outcome** | **Study or consortium** | **Participants** | **Ancestry** | **Web source** |
| --- | --- | --- | --- | --- |
| SHGB | Katherine S Ruth et al, 2020 | 425,097 individuals | European ancestry | <https://doi.org/10.1038/s41591-020-0751-5> |
| PCOS | Day, F et al, 2019 | 4,138 cases and 20,129 controls | European ancestry | [https://doi.org/10.17863/CAM.36024.](https://doi.org/10.17863/CAM.36024.) |
| PCOS | Finnegan consortium, R9 | 1,424 cases and 200,581 controls | European ancestry | <https://storage.googleapis.com/finngen-public-data-r9/summary_stats/finngen_R9_E4_PCOS.gz> |

Abbreviations: PCOS, polycystic ovary syndrome; SHBG, sex hormone binding globulin.

| **Supplementary Table 2. Associations of the genetic variants with circulating SHBG levels and their effect estimates on risk of polycystic ovary syndrome.** | | | | | | |  |
| --- | --- | --- | --- | --- | --- | --- | --- |
| **SNP** | **Chr** | **Position** | **Effect Allele** | **β** | **SE** | ***P-*value** |  |
|  |  |  |  |  |  |  |  |
| rs10788930 | 1 | 51118253 | C | -0.012 | 0.002 | 1.20E-12 |  |
| rs1110303 | 1 | 205275972 | A | -0.007 | 0.001 | 2.30E-09 |  |
| rs114165349 | 1 | 27021913 | G | 0.074 | 0.004 | 2.80E-82 |  |
| rs1211184 | 1 | 35888147 | T | 0.013 | 0.002 | 2.70E-10 |  |
| rs12136348 | 1 | 156016035 | T | -0.008 | 0.001 | 2.70E-11 |  |
| rs12138803 | 1 | 172348823 | C | 0.009 | 0.001 | 9.80E-10 |  |
| rs1223796 | 1 | 214323347 | G | 0.014 | 0.002 | 8.40E-19 |  |
| rs13303359 | 1 | 203518873 | A | -0.008 | 0.001 | 5.90E-12 |  |
| rs138755456 | 1 | 235395595 | T | -0.011 | 0.002 | 2.60E-09 |  |
| rs1762484 | 1 | 107613923 | A | 0.023 | 0.001 | 1.70E-73 |  |
| rs1870927 | 1 | 226426337 | T | -0.008 | 0.001 | 2.00E-11 |  |
| rs198358 | 1 | 11904076 | T | -0.008 | 0.001 | 9.00E-11 |  |
| rs2064074 | 1 | 171061990 | A | 0.006 | 0.001 | 3.60E-08 |  |
| rs267733 | 1 | 150958836 | A | 0.014 | 0.002 | 3.70E-21 |  |
| rs3001032 | 1 | 219727779 | T | -0.015 | 0.001 | 3.60E-29 |  |
| rs34331968 | 1 | 196659753 | T | 0.011 | 0.001 | 7.80E-20 |  |
| rs34517439 | 1 | 78450517 | C | -0.013 | 0.002 | 1.10E-12 |  |
| rs34621709 | 1 | 220980078 | C | 0.010 | 0.002 | 7.00E-11 |  |
| rs36209093 | 1 | 110229787 | C | 0.013 | 0.001 | 2.20E-18 |  |
| rs469721 | 1 | 91530001 | C | 0.011 | 0.002 | 5.40E-12 |  |
| rs61778883 | 1 | 23795177 | T | -0.013 | 0.002 | 3.30E-13 |  |
| rs630372 | 1 | 177885762 | G | -0.012 | 0.001 | 1.40E-18 |  |
| rs6541349 | 1 | 93787867 | T | -0.009 | 0.002 | 7.30E-10 |  |
| rs72663503 | 1 | 39969059 | C | 0.013 | 0.001 | 4.20E-21 |  |
| rs74090351 | 1 | 61705898 | G | -0.019 | 0.002 | 5.90E-18 |  |
| rs75077113 | 1 | 11214582 | A | -0.012 | 0.001 | 2.90E-16 |  |
| rs873308 | 1 | 25758655 | G | -0.007 | 0.001 | 6.90E-10 |  |
| rs9426829 | 1 | 154592201 | T | -0.014 | 0.001 | 1.50E-30 |  |
| rs9970073 | 1 | 214156165 | G | -0.009 | 0.001 | 6.00E-14 |  |
| rs10185143 | 2 | 25150758 | T | -0.007 | 0.001 | 2.70E-09 |  |
| rs10210970 | 2 | 28646847 | C | 0.011 | 0.002 | 1.90E-10 |  |
| rs1047891 | 2 | 211540507 | C | -0.018 | 0.001 | 1.50E-44 |  |
| rs1128249 | 2 | 165528624 | G | -0.022 | 0.001 | 1.30E-73 |  |
| rs11690748 | 2 | 48584575 | C | 0.008 | 0.001 | 9.70E-11 |  |
| rs1260326 | 2 | 27730940 | T | -0.035 | 0.001 | 1.70E-188 |  |
| rs12613243 | 2 | 111897506 | T | 0.019 | 0.003 | 7.60E-16 |  |
| rs13394092 | 2 | 85815954 | T | -0.008 | 0.002 | 1.40E-08 |  |
| rs1375045 | 2 | 112263087 | G | -0.008 | 0.001 | 1.30E-08 |  |
| rs201209127 | 2 | 227650928 | G | 0.015 | 0.002 | 7.60E-09 |  |
| rs2176040 | 2 | 227092802 | A | 0.015 | 0.001 | 2.70E-36 |  |
| rs2364717 | 2 | 178101235 | C | -0.008 | 0.001 | 2.30E-12 |  |
| rs35135293 | 2 | 20363666 | C | 0.007 | 0.001 | 1.80E-08 |  |
| rs369980015 | 2 | 165624926 | A | -0.020 | 0.004 | 1.70E-08 |  |
| rs4599150 | 2 | 191518198 | G | 0.012 | 0.001 | 8.10E-16 |  |
| rs58304838 | 2 | 208453465 | T | -0.007 | 0.001 | 3.20E-10 |  |
| rs62186584 | 2 | 241853621 | C | 0.008 | 0.001 | 5.90E-09 |  |
| rs6546096 | 2 | 64906295 | A | 0.025 | 0.001 | 3.50E-76 |  |
| rs6736913 | 2 | 42510018 | A | 0.037 | 0.004 | 3.60E-21 |  |
| rs74814151 | 2 | 70462707 | T | 0.022 | 0.003 | 9.70E-18 |  |
| rs7587219 | 2 | 227237339 | T | -0.008 | 0.001 | 2.60E-08 |  |
| rs78058190 | 2 | 219699999 | G | 0.026 | 0.003 | 7.10E-18 |  |
| rs921153 | 2 | 61563408 | G | -0.009 | 0.002 | 2.80E-10 |  |
| rs9308616 | 2 | 121340680 | T | -0.010 | 0.002 | 3.10E-10 |  |
| rs10461018 | 3 | 46995242 | C | -0.011 | 0.001 | 2.50E-19 |  |
| rs11720108 | 3 | 123069058 | C | -0.008 | 0.001 | 5.40E-11 |  |
| rs12489092 | 3 | 49550914 | G | 0.008 | 0.001 | 6.30E-11 |  |
| rs17202341 | 3 | 105452593 | A | -0.007 | 0.001 | 1.30E-09 |  |
| rs1801282 | 3 | 12393125 | C | -0.024 | 0.002 | 8.90E-43 |  |
| rs2371767 | 3 | 64718258 | G | -0.009 | 0.001 | 3.80E-11 |  |
| rs4530527 | 3 | 86800085 | A | -0.008 | 0.001 | 1.80E-09 |  |
| rs57158761 | 3 | 185371172 | A | 0.010 | 0.001 | 9.30E-18 |  |
| rs62255565 | 3 | 70701089 | C | -0.008 | 0.001 | 5.40E-09 |  |
| rs62271373 | 3 | 150066540 | T | 0.026 | 0.003 | 2.60E-25 |  |
| rs6772177 | 3 | 52497778 | C | 0.011 | 0.002 | 1.60E-12 |  |
| rs6779881 | 3 | 183358496 | C | 0.008 | 0.001 | 3.50E-08 |  |
| rs6792725 | 3 | 24520283 | A | -0.018 | 0.001 | 4.50E-43 |  |
| rs6803518 | 3 | 129735650 | T | 0.007 | 0.001 | 4.60E-08 |  |
| rs687339 | 3 | 135932359 | C | 0.031 | 0.001 | 5.30E-104 |  |
| rs784504 | 3 | 39195260 | G | -0.010 | 0.002 | 1.70E-12 |  |
| rs79287178 | 3 | 172294500 | G | 0.032 | 0.004 | 4.00E-19 |  |
| rs9849142 | 3 | 172221249 | G | -0.008 | 0.001 | 3.70E-08 |  |
| rs9849171 | 3 | 12622623 | G | -0.009 | 0.001 | 5.70E-14 |  |
| rs9872754 | 3 | 138117985 | C | 0.010 | 0.002 | 9.00E-11 |  |
| rs10027275 | 4 | 148981496 | G | 0.010 | 0.001 | 3.30E-13 |  |
| rs11097198 | 4 | 89731033 | C | 0.010 | 0.001 | 1.40E-17 |  |
| rs13108218 | 4 | 3443931 | A | 0.024 | 0.001 | 1.40E-83 |  |
| rs13150068 | 4 | 88203828 | A | 0.017 | 0.001 | 6.20E-47 |  |
| rs1433210 | 4 | 124766956 | A | -0.010 | 0.001 | 2.90E-12 |  |
| rs28636815 | 4 | 77197397 | A | -0.012 | 0.001 | 1.50E-21 |  |
| rs28712547 | 4 | 157646955 | A | -0.010 | 0.001 | 2.90E-14 |  |
| rs28925904 | 4 | 144359490 | C | 0.023 | 0.004 | 4.90E-09 |  |
| rs2970871 | 4 | 23890582 | T | 0.007 | 0.001 | 3.20E-08 |  |
| rs56257546 | 4 | 3001689 | G | -0.022 | 0.004 | 1.30E-10 |  |
| rs62294491 | 4 | 969111 | C | 0.012 | 0.002 | 3.10E-11 |  |
| rs62303689 | 4 | 56295873 | C | 0.012 | 0.002 | 3.20E-10 |  |
| rs6531735 | 4 | 39686332 | G | 0.006 | 0.001 | 1.30E-08 |  |
| rs6831257 | 4 | 100018260 | G | 0.008 | 0.001 | 3.30E-11 |  |
| rs7696472 | 4 | 69538180 | G | -0.007 | 0.001 | 4.50E-08 |  |
| rs78025076 | 4 | 110569620 | C | -0.029 | 0.004 | 1.80E-12 |  |
| rs78890745 | 4 | 159834474 | G | -0.020 | 0.002 | 2.40E-25 |  |
| rs925098 | 4 | 17919811 | G | 0.009 | 0.001 | 2.80E-09 |  |
| rs11738093 | 5 | 53301425 | A | 0.014 | 0.001 | 6.00E-24 |  |
| rs1651274 | 5 | 158020425 | A | 0.014 | 0.001 | 4.10E-22 |  |
| rs2522056 | 5 | 131801726 | G | -0.011 | 0.002 | 2.40E-11 |  |
| rs329118 | 5 | 133861663 | C | 0.010 | 0.001 | 1.40E-14 |  |
| rs40270 | 5 | 55804552 | A | 0.018 | 0.001 | 8.70E-35 |  |
| rs4976033 | 5 | 67714246 | A | 0.011 | 0.001 | 3.30E-17 |  |
| rs60681604 | 5 | 90225453 | T | -0.008 | 0.001 | 1.80E-10 |  |
| rs6556402 | 5 | 158600346 | G | -0.007 | 0.001 | 1.60E-09 |  |
| rs6860245 | 5 | 127367998 | G | -0.011 | 0.001 | 9.60E-14 |  |
| rs6879874 | 5 | 176730775 | A | -0.008 | 0.001 | 1.80E-09 |  |
| rs115447786 | 6 | 34354073 | C | 0.023 | 0.003 | 3.80E-15 |  |
| rs11967262 | 6 | 43760327 | C | 0.011 | 0.001 | 1.30E-20 |  |
| rs1202304 | 6 | 19924070 | C | -0.007 | 0.001 | 2.00E-08 |  |
| rs13200245 | 6 | 95949746 | G | -0.009 | 0.002 | 2.20E-08 |  |
| rs1738386 | 6 | 151990235 | T | -0.008 | 0.001 | 1.90E-11 |  |
| rs199607859 | 6 | 139835418 | G | -0.010 | 0.001 | 3.70E-17 |  |
| rs2208605 | 6 | 55572187 | T | -0.009 | 0.002 | 2.10E-08 |  |
| rs2299055 | 6 | 15398331 | A | 0.011 | 0.002 | 2.20E-08 |  |
| rs2523570 | 6 | 31329734 | C | 0.014 | 0.001 | 2.30E-21 |  |
| rs28360642 | 6 | 41667506 | A | 0.020 | 0.002 | 5.80E-36 |  |
| rs4709746 | 6 | 164133001 | C | -0.012 | 0.002 | 4.50E-11 |  |
| rs555754 | 6 | 160769423 | G | -0.018 | 0.001 | 1.70E-59 |  |
| rs58321169 | 6 | 126868567 | C | 0.010 | 0.001 | 5.60E-14 |  |
| rs6941110 | 6 | 34791334 | C | -0.015 | 0.001 | 3.10E-34 |  |
| rs72959041 | 6 | 127454893 | G | 0.023 | 0.003 | 1.60E-16 |  |
| rs76230335 | 6 | 32131667 | G | 0.024 | 0.004 | 9.30E-11 |  |
| rs7756992 | 6 | 20679709 | A | 0.010 | 0.001 | 2.30E-14 |  |
| rs7762391 | 6 | 117507521 | A | 0.006 | 0.001 | 3.20E-08 |  |
| rs9379084 | 6 | 7231843 | G | 0.013 | 0.002 | 1.50E-10 |  |
| rs10238028 | 7 | 99208899 | A | -0.014 | 0.002 | 6.50E-09 |  |
| rs10486782 | 7 | 15884421 | A | 0.008 | 0.001 | 2.50E-08 |  |
| rs114949263 | 7 | 150498245 | T | -0.014 | 0.002 | 2.70E-14 |  |
| rs12673873 | 7 | 26379388 | C | 0.011 | 0.002 | 9.90E-11 |  |
| rs13237750 | 7 | 46456878 | C | 0.016 | 0.003 | 1.60E-09 |  |
| rs1688606 | 7 | 97974851 | G | -0.038 | 0.002 | 7.50E-135 |  |
| rs17471520 | 7 | 27178790 | T | -0.012 | 0.002 | 2.00E-08 |  |
| rs17492269 | 7 | 70047405 | G | 0.010 | 0.002 | 2.80E-09 |  |
| rs17884589 | 7 | 100493711 | G | -0.017 | 0.002 | 4.30E-29 |  |
| rs2051748 | 7 | 93199574 | A | -0.007 | 0.001 | 2.00E-08 |  |
| rs2551774 | 7 | 135119417 | A | 0.007 | 0.001 | 1.80E-09 |  |
| rs34706862 | 7 | 129678625 | C | -0.008 | 0.001 | 1.60E-09 |  |
| rs4731702 | 7 | 130433384 | C | -0.011 | 0.001 | 5.30E-21 |  |
| rs57193069 | 7 | 1862417 | A | -0.009 | 0.001 | 1.80E-12 |  |
| rs587736602 | 7 | 74332365 | C | -0.011 | 0.002 | 4.20E-09 |  |
| rs702814 | 7 | 28172732 | C | -0.012 | 0.001 | 1.50E-24 |  |
| rs73670309 | 7 | 1065947 | C | 0.012 | 0.002 | 1.10E-10 |  |
| rs76200127 | 7 | 81533257 | G | -0.017 | 0.002 | 5.90E-15 |  |
| rs848476 | 7 | 77541673 | G | 0.010 | 0.001 | 1.10E-16 |  |
| rs10095930 | 8 | 116974302 | C | 0.010 | 0.001 | 1.10E-15 |  |
| rs10096213 | 8 | 36853213 | C | 0.012 | 0.002 | 7.50E-13 |  |
| rs111267789 | 8 | 81872046 | C | -0.011 | 0.002 | 9.20E-09 |  |
| rs11774700 | 8 | 118220270 | T | -0.008 | 0.001 | 1.50E-09 |  |
| rs12543287 | 8 | 42334511 | G | -0.011 | 0.001 | 1.80E-18 |  |
| rs12680692 | 8 | 12618225 | T | 0.007 | 0.001 | 1.10E-08 |  |
| rs13277751 | 8 | 59415337 | T | -0.009 | 0.001 | 4.20E-13 |  |
| rs150539196 | 8 | 81399180 | A | -0.050 | 0.003 | 3.30E-52 |  |
| rs1985022 | 8 | 145684031 | C | -0.009 | 0.001 | 1.90E-15 |  |
| rs2954032 | 8 | 126493392 | A | -0.010 | 0.001 | 8.80E-14 |  |
| rs440837 | 8 | 81461974 | A | -0.025 | 0.002 | 1.70E-67 |  |
| rs59241933 | 8 | 72521293 | C | 0.009 | 0.001 | 4.10E-11 |  |
| rs62515125 | 8 | 81734403 | C | -0.022 | 0.004 | 5.90E-09 |  |
| rs6991364 | 8 | 145050345 | G | -0.008 | 0.001 | 8.60E-12 |  |
| rs7822342 | 8 | 81246699 | A | -0.010 | 0.001 | 3.80E-18 |  |
| rs9644032 | 8 | 23414822 | T | 0.008 | 0.001 | 1.20E-11 |  |
| rs9987289 | 8 | 9183358 | A | -0.022 | 0.002 | 1.40E-26 |  |
| rs10811662 | 9 | 22134253 | G | -0.009 | 0.002 | 2.10E-08 |  |
| rs10959107 | 9 | 1054991 | G | 0.010 | 0.002 | 2.80E-11 |  |
| rs10961205 | 9 | 13722479 | G | -0.007 | 0.001 | 4.60E-08 |  |
| rs2004354 | 9 | 123487903 | A | 0.010 | 0.001 | 1.10E-13 |  |
| rs2986670 | 9 | 113190709 | C | 0.009 | 0.001 | 7.70E-11 |  |
| rs35233014 | 9 | 137268177 | C | 0.015 | 0.001 | 3.80E-27 |  |
| rs4743772 | 9 | 107724990 | C | 0.008 | 0.001 | 8.60E-09 |  |
| rs4876993 | 9 | 92281403 | T | 0.007 | 0.001 | 2.50E-09 |  |
| rs568656 | 9 | 4133874 | A | -0.012 | 0.001 | 4.40E-18 |  |
| rs62580766 | 9 | 113034490 | C | -0.011 | 0.002 | 7.50E-16 |  |
| rs696825 | 9 | 86583076 | C | -0.024 | 0.001 | 4.50E-63 |  |
| rs80126506 | 9 | 137159427 | G | -0.007 | 0.001 | 2.10E-10 |  |
| rs820504 | 9 | 6668278 | G | 0.014 | 0.002 | 2.20E-16 |  |
| rs9697210 | 9 | 131468740 | G | 0.015 | 0.002 | 9.00E-20 |  |
| rs10995445 | 10 | 64874754 | A | -0.038 | 0.001 | 1.00E-200 |  |
| rs11599530 | 10 | 122996499 | C | -0.007 | 0.001 | 2.90E-09 |  |
| rs1414929 | 10 | 95328816 | A | 0.009 | 0.002 | 1.30E-08 |  |
| rs1782652 | 10 | 81074125 | T | 0.014 | 0.001 | 1.20E-26 |  |
| rs2068888 | 10 | 94839642 | G | -0.013 | 0.001 | 2.00E-26 |  |
| rs2915023 | 10 | 77282899 | A | -0.011 | 0.002 | 3.90E-08 |  |
| rs35198068 | 10 | 114754784 | T | 0.011 | 0.001 | 2.00E-19 |  |
| rs6415872 | 10 | 63660689 | G | 0.009 | 0.001 | 6.40E-17 |  |
| rs72812190 | 10 | 70989210 | C | 0.011 | 0.002 | 9.40E-14 |  |
| rs7477114 | 10 | 5252838 | G | 0.018 | 0.002 | 1.30E-30 |  |
| rs79780963 | 10 | 104952499 | C | -0.015 | 0.002 | 3.50E-10 |  |
| rs899865 | 10 | 36473044 | T | 0.007 | 0.001 | 1.00E-08 |  |
| rs915506 | 10 | 97805074 | G | -0.009 | 0.001 | 2.10E-14 |  |
| rs1037169 | 11 | 13361005 | T | 0.013 | 0.001 | 1.20E-24 |  |
| rs10892468 | 11 | 119676471 | T | -0.007 | 0.001 | 6.40E-09 |  |
| rs10893876 | 11 | 128353007 | C | 0.008 | 0.001 | 3.50E-08 |  |
| rs11021232 | 11 | 95320808 | T | 0.013 | 0.002 | 4.00E-17 |  |
| rs11602707 | 11 | 102045643 | C | 0.008 | 0.001 | 2.00E-09 |  |
| rs12804411 | 11 | 69284200 | C | -0.014 | 0.001 | 2.60E-24 |  |
| rs174537 | 11 | 61552680 | G | 0.012 | 0.001 | 5.50E-21 |  |
| rs2074310 | 11 | 17421886 | T | -0.008 | 0.001 | 5.30E-09 |  |
| rs2276133 | 11 | 65562257 | G | -0.011 | 0.001 | 9.00E-17 |  |
| rs2280231 | 11 | 47600438 | C | 0.007 | 0.001 | 7.50E-09 |  |
| rs2351958 | 11 | 16248020 | C | 0.011 | 0.001 | 6.30E-22 |  |
| rs2924545 | 11 | 68883281 | G | 0.008 | 0.001 | 5.50E-13 |  |
| rs3842763 | 11 | 2179204 | G | -0.011 | 0.001 | 6.80E-16 |  |
| rs7481219 | 11 | 307808 | A | 0.008 | 0.001 | 3.50E-10 |  |
| rs76491020 | 11 | 32673898 | G | -0.012 | 0.002 | 1.30E-09 |  |
| rs1043763 | 12 | 122630909 | G | -0.010 | 0.002 | 5.00E-13 |  |
| rs10774095 | 12 | 3125648 | G | -0.009 | 0.002 | 7.40E-09 |  |
| rs11045265 | 12 | 20614084 | T | -0.010 | 0.001 | 2.70E-12 |  |
| rs11064372 | 12 | 6868590 | C | 0.007 | 0.001 | 2.00E-09 |  |
| rs11110390 | 12 | 100874901 | C | 0.012 | 0.001 | 2.60E-22 |  |
| rs11169199 | 12 | 50280430 | T | -0.008 | 0.001 | 2.80E-10 |  |
| rs11172134 | 12 | 57645789 | T | -0.016 | 0.002 | 9.50E-26 |  |
| rs12311848 | 12 | 124486851 | A | -0.014 | 0.001 | 1.60E-29 |  |
| rs12320328 | 12 | 25408464 | A | 0.022 | 0.002 | 8.50E-28 |  |
| rs17029952 | 12 | 100448921 | T | 0.035 | 0.005 | 1.30E-15 |  |
| rs4149056 | 12 | 21331549 | T | 0.030 | 0.002 | 1.50E-74 |  |
| rs4307773 | 12 | 51144432 | T | 0.014 | 0.001 | 5.90E-31 |  |
| rs4759319 | 12 | 54424731 | G | -0.007 | 0.001 | 4.90E-09 |  |
| rs4764939 | 12 | 103522952 | C | 0.024 | 0.001 | 6.70E-91 |  |
| rs5009837 | 12 | 102810269 | T | 0.012 | 0.001 | 4.20E-20 |  |
| rs61934152 | 12 | 93603336 | T | 0.012 | 0.002 | 5.20E-13 |  |
| rs7304705 | 12 | 111438001 | A | -0.028 | 0.002 | 4.20E-38 |  |
| rs7310409 | 12 | 121424861 | A | -0.013 | 0.001 | 1.00E-26 |  |
| rs740893 | 12 | 676209 | C | -0.009 | 0.002 | 5.00E-11 |  |
| rs76895963 | 12 | 4384844 | T | -0.075 | 0.005 | 1.30E-64 |  |
| rs7968902 | 12 | 66363070 | T | 0.007 | 0.001 | 2.40E-09 |  |
| rs1239945 | 13 | 51107757 | G | 0.009 | 0.001 | 4.90E-12 |  |
| rs2812208 | 13 | 50707087 | G | -0.035 | 0.004 | 1.50E-18 |  |
| rs7321688 | 13 | 115000365 | C | 0.009 | 0.001 | 1.70E-09 |  |
| rs9556403 | 13 | 95236825 | A | -0.007 | 0.001 | 6.80E-10 |  |
| rs11621792 | 14 | 24871926 | C | 0.026 | 0.001 | 4.00E-102 |  |
| rs12717441 | 14 | 23721960 | C | -0.013 | 0.002 | 3.70E-18 |  |
| rs13379043 | 14 | 74250126 | T | -0.011 | 0.001 | 2.20E-16 |  |
| rs17580 | 14 | 94847262 | T | -0.024 | 0.003 | 8.90E-18 |  |
| rs2239222 | 14 | 73011885 | A | -0.010 | 0.001 | 2.10E-16 |  |
| rs2498786 | 14 | 105262368 | C | 0.011 | 0.001 | 5.50E-19 |  |
| rs28929474 | 14 | 94844947 | C | -0.061 | 0.004 | 3.10E-43 |  |
| rs72683923 | 14 | 50735947 | T | -0.027 | 0.004 | 1.60E-11 |  |
| rs11635675 | 15 | 63793238 | T | 0.016 | 0.001 | 5.10E-39 |  |
| rs11637595 | 15 | 40387728 | C | 0.012 | 0.001 | 2.40E-18 |  |
| rs139974673 | 15 | 44027885 | T | 0.054 | 0.004 | 1.40E-49 |  |
| rs16976170 | 15 | 96651048 | G | -0.029 | 0.004 | 1.80E-14 |  |
| rs2218181 | 15 | 66872325 | T | 0.009 | 0.001 | 4.10E-14 |  |
| rs2263551 | 15 | 40979543 | G | 0.007 | 0.001 | 3.70E-09 |  |
| rs56332871 | 15 | 96714816 | C | -0.039 | 0.001 | 9.20E-188 |  |
| rs62023531 | 15 | 53168857 | C | 0.021 | 0.002 | 2.10E-28 |  |
| rs79237700 | 15 | 53741612 | T | 0.020 | 0.003 | 7.70E-11 |  |
| rs79391862 | 15 | 53739426 | A | 0.073 | 0.005 | 1.70E-46 |  |
| rs8033077 | 15 | 35266501 | C | 0.009 | 0.001 | 3.10E-11 |  |
| rs11641834 | 16 | 88070573 | C | 0.010 | 0.001 | 3.10E-17 |  |
| rs1684608 | 16 | 4676852 | C | 0.009 | 0.002 | 1.50E-10 |  |
| rs28429148 | 16 | 53798319 | G | -0.006 | 0.001 | 1.00E-08 |  |
| rs2925979 | 16 | 81534790 | T | -0.014 | 0.001 | 1.00E-26 |  |
| rs4122352 | 16 | 15174571 | G | -0.011 | 0.001 | 1.80E-16 |  |
| rs67890964 | 16 | 83979317 | T | -0.011 | 0.001 | 7.00E-19 |  |
| rs72782727 | 16 | 11878033 | G | 0.009 | 0.001 | 2.30E-11 |  |
| rs749671 | 16 | 31088347 | G | 0.008 | 0.001 | 1.40E-11 |  |
| rs77147683 | 16 | 71623594 | C | -0.008 | 0.001 | 8.80E-10 |  |
| rs10153315 | 17 | 79481772 | T | 0.009 | 0.001 | 7.50E-16 |  |
| rs10401031 | 17 | 73799110 | A | -0.010 | 0.001 | 4.20E-11 |  |
| rs113201977 | 17 | 47298215 | A | 0.028 | 0.002 | 2.50E-72 |  |
| rs118098353 | 17 | 7531244 | C | -0.107 | 0.005 | 1.70E-129 |  |
| rs139725501 | 17 | 7426907 | C | -0.079 | 0.003 | 1.00E-200 |  |
| rs140302625 | 17 | 47379867 | G | -0.062 | 0.002 | 4.20E-193 |  |
| rs141667726 | 17 | 7244137 | C | 0.286 | 0.007 | 1.00E-200 |  |
| rs144989856 | 17 | 7528097 | G | 0.074 | 0.007 | 9.00E-30 |  |
| rs146876556 | 17 | 7391376 | G | -0.103 | 0.008 | 3.80E-36 |  |
| rs1642792 | 17 | 7576151 | G | 0.080 | 0.006 | 6.90E-49 |  |
| rs17880847 | 17 | 7573897 | T | 0.104 | 0.005 | 1.90E-89 |  |
| rs181975550 | 17 | 7595379 | C | -0.055 | 0.004 | 2.30E-55 |  |
| rs183889114 | 17 | 7545273 | C | -0.032 | 0.005 | 1.30E-11 |  |
| rs1889014 | 17 | 17767165 | C | -0.015 | 0.001 | 4.00E-37 |  |
| rs35981831 | 17 | 47303222 | T | 0.025 | 0.004 | 5.50E-11 |  |
| rs575383031 | 17 | 7368399 | T | 0.060 | 0.005 | 2.40E-31 |  |
| rs59368120 | 17 | 38257828 | A | 0.021 | 0.003 | 5.20E-15 |  |
| rs62062617 | 17 | 7658034 | C | -0.041 | 0.003 | 3.90E-56 |  |
| rs7217661 | 17 | 65266562 | T | 0.007 | 0.001 | 7.70E-10 |  |
| rs72844546 | 17 | 73149850 | C | 0.010 | 0.001 | 3.10E-18 |  |
| rs77142415 | 17 | 45752738 | G | 0.018 | 0.001 | 8.10E-54 |  |
| rs79693490 | 17 | 7502608 | G | 0.168 | 0.007 | 3.50E-146 |  |
| rs8178824 | 17 | 64224775 | C | 0.043 | 0.004 | 9.40E-36 |  |
| rs11664106 | 18 | 2846812 | A | -0.008 | 0.001 | 3.60E-10 |  |
| rs4092465 | 18 | 55080437 | A | -0.012 | 0.001 | 3.60E-20 |  |
| rs4327143 | 18 | 71925113 | G | -0.009 | 0.001 | 1.70E-11 |  |
| rs7233512 | 18 | 42595076 | G | 0.008 | 0.001 | 2.10E-09 |  |
| rs7240326 | 18 | 60917351 | T | -0.009 | 0.001 | 1.90E-12 |  |
| rs1076448 | 19 | 2792343 | G | -0.021 | 0.001 | 1.40E-60 |  |
| rs11668201 | 19 | 59003632 | A | -0.009 | 0.002 | 1.00E-08 |  |
| rs140965448 | 19 | 37690941 | G | 0.016 | 0.003 | 3.70E-08 |  |
| rs16996148 | 19 | 19658472 | G | 0.012 | 0.002 | 1.20E-08 |  |
| rs2018519 | 19 | 35559787 | T | -0.020 | 0.002 | 3.40E-40 |  |
| rs202200760 | 19 | 17346854 | G | -0.076 | 0.003 | 5.20E-120 |  |
| rs2115107 | 19 | 7968168 | G | 0.008 | 0.001 | 5.10E-12 |  |
| rs273510 | 19 | 18223350 | A | 0.010 | 0.001 | 1.90E-13 |  |
| rs28435282 | 19 | 48119969 | C | -0.008 | 0.001 | 5.80E-14 |  |
| rs34255979 | 19 | 46384830 | C | -0.028 | 0.002 | 7.90E-52 |  |
| rs4804416 | 19 | 7223848 | T | 0.018 | 0.001 | 9.20E-54 |  |
| rs551265709 | 19 | 17425953 | C | -0.016 | 0.003 | 1.30E-08 |  |
| rs59774409 | 19 | 50016748 | C | -0.018 | 0.002 | 5.20E-16 |  |
| rs60018147 | 19 | 3375572 | A | -0.016 | 0.002 | 6.10E-15 |  |
| rs7250869 | 19 | 33887405 | T | -0.011 | 0.001 | 2.70E-20 |  |
| rs11167234 | 20 | 32983619 | T | -0.010 | 0.001 | 1.70E-15 |  |
| rs13042148 | 20 | 32298286 | C | 0.016 | 0.002 | 1.90E-21 |  |
| rs16995626 | 20 | 49540925 | T | -0.018 | 0.002 | 3.70E-15 |  |
| rs1741344 | 20 | 4101800 | C | -0.007 | 0.001 | 3.40E-09 |  |
| rs2618566 | 20 | 17844684 | G | 0.009 | 0.001 | 1.80E-13 |  |
| rs4810580 | 20 | 45594295 | T | 0.010 | 0.002 | 6.20E-10 |  |
| rs6062344 | 20 | 62696024 | C | -0.007 | 0.001 | 3.70E-10 |  |
| rs6073431 | 20 | 43040569 | C | -0.017 | 0.001 | 6.20E-43 |  |
| rs28412679 | 22 | 50730320 | T | -0.007 | 0.001 | 3.90E-09 |  |
| rs3747207 | 22 | 44324855 | G | -0.017 | 0.002 | 1.00E-30 |  |
| rs5753111 | 22 | 30779211 | T | 0.013 | 0.001 | 4.40E-23 |  |
| rs695272 | 22 | 28856760 | T | 0.009 | 0.001 | 8.70E-12 |  |
| rs763263 | 22 | 42315790 | C | -0.009 | 0.002 | 5.9E-09 |  |

Abbreviations: SHBG, sex hormone binding globulin; PCOS, polycystic ovary syndrome; Chr, chromosome; SE, standard error; SNP, single nucleotide polymorphism.

**Supplementary Table 3.** Associations of the genetic variants with SHBG levels adjusted by BMI and their effects on polycystic ovary syndrome.

| **SNP** | **Chr** | **Position** | **Effect Allele** | **β** | **SE** | ***P-***value |
| --- | --- | --- | --- | --- | --- | --- |
|  |  |  |  |  |  |  |
| rs10753556 | 1 | 23732762 | A | -0.010 | 0.001 | 2.30E-16 |
| rs10797877 | 1 | 171075556 | C | 0.006 | 0.001 | 1.20E-15 |
| rs114165349 | 1 | 27021913 | G | 0.081 | 0.003 | 1.00E-200 |
| rs1172128 | 1 | 205245233 | A | 0.011 | 0.001 | 9.40E-15 |
| rs12078447 | 1 | 51499525 | A | -0.011 | 0.001 | 9.20E-18 |
| rs12138803 | 1 | 172348823 | C | 0.006 | 0.001 | 2.30E-10 |
| rs1223807 | 1 | 214356703 | A | 0.015 | 0.001 | 3.30E-44 |
| rs12385720 | 1 | 93539383 | G | 0.007 | 0.001 | 6.60E-19 |
| rs141845046 | 1 | 154987704 | C | -0.015 | 0.003 | 1.10E-08 |
| rs146199089 | 1 | 150314457 | G | -0.024 | 0.003 | 2.60E-13 |
| rs16842246 | 1 | 197982013 | T | -0.012 | 0.002 | 2.90E-12 |
| rs1730859 | 1 | 107617707 | G | 0.026 | 0.001 | 6.60E-197 |
| rs1870927 | 1 | 226426337 | T | -0.007 | 0.001 | 3.70E-18 |
| rs2107518 | 1 | 93247835 | C | -0.006 | 0.001 | 3.00E-11 |
| rs213494 | 1 | 54877103 | C | 0.006 | 0.001 | 7.90E-12 |
| rs2247213 | 1 | 221055463 | G | 0.012 | 0.001 | 1.10E-45 |
| rs267733 | 1 | 150958836 | A | 0.012 | 0.001 | 6.60E-29 |
| rs2755263 | 1 | 67529243 | A | -0.005 | 0.001 | 3.80E-08 |
| rs2791644 | 1 | 11160674 | C | 0.009 | 0.001 | 2.40E-21 |
| rs2802770 | 1 | 203518456 | T | -0.007 | 0.001 | 5.90E-15 |
| rs28549287 | 1 | 110230138 | G | 0.013 | 0.001 | 5.00E-35 |
| rs3001032 | 1 | 219727779 | T | -0.011 | 0.001 | 1.30E-37 |
| rs34517439 | 1 | 78450517 | C | -0.009 | 0.001 | 8.20E-13 |
| rs35346083 | 1 | 25788425 | C | 0.008 | 0.001 | 4.80E-24 |
| rs36086195 | 1 | 16510894 | C | -0.011 | 0.001 | 2.90E-43 |
| rs3768321 | 1 | 40035928 | G | 0.013 | 0.001 | 4.40E-35 |
| rs4310395 | 1 | 232795105 | A | -0.004 | 0.001 | 1.70E-08 |
| rs4639796 | 1 | 197126649 | G | 0.012 | 0.001 | 4.30E-28 |
| rs469882 | 1 | 91530432 | A | 0.009 | 0.001 | 9.10E-17 |
| rs559986 | 1 | 35841258 | C | 0.012 | 0.001 | 6.60E-17 |
| rs59708846 | 1 | 61687651 | G | -0.019 | 0.002 | 1.60E-37 |
| rs61830291 | 1 | 221001142 | A | -0.009 | 0.001 | 1.20E-10 |
| rs670323 | 1 | 177868990 | G | -0.010 | 0.001 | 1.00E-24 |
| rs6703810 | 1 | 39345358 | T | -0.010 | 0.002 | 4.20E-10 |
| rs7515496 | 1 | 235408221 | G | -0.010 | 0.001 | 6.30E-17 |
| rs7539725 | 1 | 10125407 | A | -0.007 | 0.001 | 8.00E-10 |
| rs7542169 | 1 | 166864373 | A | 0.006 | 0.001 | 2.10E-11 |
| rs79940514 | 1 | 61914031 | G | 0.010 | 0.002 | 4.80E-11 |
| rs9427104 | 1 | 154589232 | C | -0.012 | 0.001 | 8.60E-53 |
| rs9970073 | 1 | 214156165 | G | -0.007 | 0.001 | 3.10E-18 |
| rs10169561 | 2 | 180458771 | T | -0.005 | 0.001 | 6.30E-09 |
| rs10188870 | 2 | 191061633 | G | 0.005 | 0.001 | 2.80E-08 |
| rs10202868 | 2 | 191563991 | T | 0.011 | 0.001 | 7.10E-26 |
| rs10210970 | 2 | 28646847 | C | 0.009 | 0.001 | 7.90E-15 |
| rs1047891 | 2 | 211540507 | C | -0.010 | 0.001 | 1.20E-29 |
| rs11164095 | 2 | 97155208 | C | -0.006 | 0.001 | 4.10E-10 |
| rs11685829 | 2 | 234340872 | T | -0.006 | 0.001 | 1.00E-13 |
| rs11690176 | 2 | 242356887 | G | -0.005 | 0.001 | 1.30E-08 |
| rs11690748 | 2 | 48584575 | C | 0.006 | 0.001 | 7.30E-15 |
| rs13389219 | 2 | 165528876 | C | -0.016 | 0.001 | 1.60E-88 |
| rs1375045 | 2 | 112263087 | G | -0.007 | 0.001 | 6.80E-16 |
| rs143853610 | 2 | 39258838 | C | -0.022 | 0.004 | 2.20E-08 |
| rs144966294 | 2 | 227650629 | T | 0.012 | 0.002 | 2.60E-10 |
| rs16830920 | 2 | 135465466 | A | 0.007 | 0.001 | 5.80E-09 |
| rs17008851 | 2 | 61606097 | A | -0.010 | 0.001 | 2.10E-16 |
| rs185241389 | 2 | 112024305 | C | -0.008 | 0.001 | 3.00E-10 |
| rs2004349 | 2 | 219289643 | A | -0.009 | 0.001 | 1.30E-24 |
| rs2307394 | 2 | 148716428 | T | -0.006 | 0.001 | 7.10E-12 |
| rs2364717 | 2 | 178101235 | C | -0.006 | 0.001 | 3.30E-16 |
| rs2542522 | 2 | 71609785 | G | -0.007 | 0.001 | 1.90E-08 |
| rs2943641 | 2 | 227093745 | T | 0.014 | 0.001 | 9.80E-66 |
| rs35633876 | 2 | 20363074 | G | 0.008 | 0.001 | 2.90E-23 |
| rs35676483 | 2 | 223540323 | T | -0.007 | 0.001 | 1.10E-08 |
| rs3761706 | 2 | 111899881 | G | 0.018 | 0.002 | 9.80E-28 |
| rs4128205 | 2 | 165467068 | A | 0.005 | 0.001 | 4.10E-12 |
| rs4665710 | 2 | 21221035 | A | -0.006 | 0.001 | 4.60E-09 |
| rs4665972 | 2 | 27598097 | T | -0.037 | 0.001 | 1.00E-200 |
| rs4668732 | 2 | 11716919 | A | -0.006 | 0.001 | 2.90E-13 |
| rs57467915 | 2 | 220081416 | G | 0.022 | 0.003 | 3.90E-11 |
| rs58304838 | 2 | 208453465 | T | -0.007 | 0.001 | 1.70E-18 |
| rs6546096 | 2 | 64906295 | A | 0.021 | 0.001 | 3.00E-110 |
| rs6727165 | 2 | 43091163 | G | -0.004 | 0.001 | 3.30E-08 |
| rs6736913 | 2 | 42510018 | A | 0.032 | 0.003 | 2.30E-32 |
| rs6742215 | 2 | 65690496 | A | 0.006 | 0.001 | 2.70E-13 |
| rs6758199 | 2 | 70537173 | C | 0.018 | 0.002 | 1.10E-28 |
| rs72798731 | 2 | 32515337 | C | -0.014 | 0.002 | 7.20E-09 |
| rs73139123 | 2 | 630075 | C | -0.006 | 0.001 | 2.80E-08 |
| rs7557491 | 2 | 64852557 | C | -0.006 | 0.001 | 1.40E-08 |
| rs9677933 | 2 | 121337280 | C | -0.007 | 0.001 | 1.80E-10 |
| rs11130982 | 3 | 64728312 | T | 0.005 | 0.001 | 1.40E-08 |
| rs12487736 | 3 | 47459679 | C | 0.009 | 0.001 | 3.90E-27 |
| rs12636106 | 3 | 122164156 | C | -0.006 | 0.001 | 1.60E-08 |
| rs12695685 | 3 | 138096097 | A | 0.008 | 0.001 | 5.50E-12 |
| rs13075010 | 3 | 56585975 | T | 0.005 | 0.001 | 6.10E-12 |
| rs13082048 | 3 | 86964322 | A | 0.006 | 0.001 | 2.60E-10 |
| rs13315174 | 3 | 105406468 | G | 0.008 | 0.001 | 6.00E-17 |
| rs1456330 | 3 | 183400967 | A | 0.007 | 0.001 | 3.10E-12 |
| rs1801282 | 3 | 12393125 | C | -0.022 | 0.001 | 3.00E-67 |
| rs1965132 | 3 | 69147519 | C | 0.005 | 0.001 | 1.90E-10 |
| rs2176887 | 3 | 101105268 | C | -0.009 | 0.002 | 2.70E-09 |
| rs2960420 | 3 | 12314512 | C | 0.006 | 0.001 | 8.10E-13 |
| rs34970607 | 3 | 123090360 | G | -0.007 | 0.001 | 2.20E-14 |
| rs36012032 | 3 | 52814709 | C | -0.011 | 0.001 | 2.00E-14 |
| rs55735727 | 3 | 169488148 | A | -0.007 | 0.001 | 2.60E-13 |
| rs57158761 | 3 | 185371172 | A | 0.009 | 0.001 | 1.70E-27 |
| rs62271373 | 3 | 150066540 | T | 0.018 | 0.002 | 2.30E-24 |
| rs62292896 | 3 | 132186187 | G | 0.006 | 0.001 | 3.40E-08 |
| rs6777420 | 3 | 142002869 | C | -0.007 | 0.001 | 8.80E-09 |
| rs6792725 | 3 | 24520283 | A | -0.015 | 0.001 | 1.10E-63 |
| rs687339 | 3 | 135932359 | C | 0.027 | 0.001 | 1.30E-175 |
| rs7430950 | 3 | 196233136 | A | 0.006 | 0.001 | 3.60E-11 |
| rs7628382 | 3 | 70641810 | A | 0.005 | 0.001 | 8.20E-09 |
| rs77395777 | 3 | 24372670 | C | 0.017 | 0.003 | 1.20E-09 |
| rs784504 | 3 | 39195260 | G | -0.007 | 0.001 | 4.00E-14 |
| rs79287178 | 3 | 172294500 | G | 0.037 | 0.003 | 1.70E-51 |
| rs9823118 | 3 | 129721303 | G | -0.006 | 0.001 | 6.10E-11 |
| rs9849142 | 3 | 172221249 | G | -0.006 | 0.001 | 6.80E-09 |
| rs9849171 | 3 | 12622623 | G | -0.009 | 0.001 | 4.10E-25 |
| rs10027275 | 4 | 148981496 | G | 0.011 | 0.001 | 5.70E-36 |
| rs114053844 | 4 | 110465025 | T | -0.028 | 0.003 | 6.20E-19 |
| rs11721999 | 4 | 185377222 | G | 0.007 | 0.001 | 9.90E-11 |
| rs11729169 | 4 | 171019391 | C | -0.011 | 0.001 | 2.80E-15 |
| rs13108218 | 4 | 3443931 | A | 0.024 | 0.001 | 3.00E-172 |
| rs13137857 | 4 | 149619413 | C | -0.005 | 0.001 | 4.40E-08 |
| rs13150068 | 4 | 88203828 | A | 0.018 | 0.001 | 1.40E-112 |
| rs2073503 | 4 | 3536176 | C | -0.006 | 0.001 | 5.00E-12 |
| rs2602856 | 4 | 100029145 | C | 0.013 | 0.001 | 2.30E-55 |
| rs2707450 | 4 | 17942560 | C | 0.007 | 0.001 | 8.20E-14 |
| rs28418580 | 4 | 89742244 | C | 0.007 | 0.001 | 7.20E-14 |
| rs28473232 | 4 | 37406477 | A | -0.006 | 0.001 | 2.60E-09 |
| rs28636815 | 4 | 77197397 | A | -0.013 | 0.001 | 1.20E-51 |
| rs28925904 | 4 | 144359490 | C | 0.021 | 0.003 | 3.60E-16 |
| rs2970877 | 4 | 23887454 | T | -0.009 | 0.001 | 1.50E-20 |
| rs3097924 | 4 | 124705993 | C | -0.005 | 0.001 | 1.70E-09 |
| rs34052949 | 4 | 88392585 | A | 0.007 | 0.001 | 1.60E-11 |
| rs34707604 | 4 | 69491456 | T | -0.016 | 0.001 | 7.30E-64 |
| rs376263969 | 4 | 966342 | A | 0.011 | 0.002 | 8.80E-09 |
| rs4691375 | 4 | 157650930 | A | -0.009 | 0.001 | 1.20E-23 |
| rs56257546 | 4 | 3001689 | G | -0.020 | 0.002 | 1.20E-18 |
| rs6531735 | 4 | 39686332 | G | 0.005 | 0.001 | 1.00E-09 |
| rs6848144 | 4 | 104175964 | T | -0.005 | 0.001 | 2.50E-08 |
| rs6848998 | 4 | 128989327 | G | -0.007 | 0.001 | 4.00E-15 |
| rs73193388 | 4 | 3401678 | C | -0.010 | 0.001 | 5.20E-18 |
| rs7678138 | 4 | 120106766 | G | 0.011 | 0.001 | 1.70E-18 |
| rs7696472 | 4 | 69538180 | G | -0.013 | 0.001 | 8.10E-58 |
| rs78890745 | 4 | 159834474 | G | -0.017 | 0.001 | 1.70E-37 |
| rs819203 | 4 | 56257103 | A | 0.005 | 0.001 | 2.00E-09 |
| rs10041660 | 5 | 122650224 | T | -0.005 | 0.001 | 2.00E-10 |
| rs11957006 | 5 | 156802909 | C | -0.011 | 0.002 | 2.10E-09 |
| rs138373837 | 5 | 36219710 | C | -0.018 | 0.003 | 4.00E-11 |
| rs138611541 | 5 | 56228040 | T | 0.011 | 0.002 | 6.50E-13 |
| rs152439 | 5 | 141924395 | T | 0.009 | 0.002 | 8.10E-09 |
| rs2431752 | 5 | 162882702 | G | -0.010 | 0.001 | 3.50E-14 |
| rs2522064 | 5 | 131806488 | G | -0.010 | 0.001 | 2.80E-20 |
| rs2703636 | 5 | 72922647 | C | -0.006 | 0.001 | 2.00E-11 |
| rs2914233 | 5 | 158013856 | T | 0.012 | 0.001 | 5.30E-31 |
| rs329120 | 5 | 133861756 | C | 0.009 | 0.001 | 2.30E-27 |
| rs40270 | 5 | 55804552 | A | 0.016 | 0.001 | 2.30E-60 |
| rs4073358 | 5 | 112382137 | C | -0.005 | 0.001 | 2.10E-08 |
| rs4266430 | 5 | 38749736 | C | -0.005 | 0.001 | 1.50E-09 |
| rs4976033 | 5 | 67714246 | A | 0.007 | 0.001 | 9.70E-20 |
| rs58729412 | 5 | 90169070 | T | -0.007 | 0.001 | 1.70E-13 |
| rs6449592 | 5 | 61538487 | A | 0.005 | 0.001 | 1.20E-11 |
| rs6860245 | 5 | 127367998 | G | -0.011 | 0.001 | 3.20E-30 |
| rs72643433 | 5 | 158364449 | G | -0.006 | 0.001 | 1.60E-11 |
| rs72709458 | 5 | 1283755 | C | 0.006 | 0.001 | 1.50E-09 |
| rs72753349 | 5 | 57392079 | C | -0.011 | 0.002 | 2.90E-08 |
| rs75049939 | 5 | 173334219 | T | 0.007 | 0.001 | 7.20E-18 |
| rs7735249 | 5 | 53310139 | C | 0.019 | 0.001 | 3.90E-46 |
| rs11155787 | 6 | 151686905 | C | 0.006 | 0.001 | 1.10E-12 |
| rs11967262 | 6 | 43760327 | C | 0.009 | 0.001 | 1.50E-30 |
| rs12661232 | 6 | 130379160 | T | 0.011 | 0.001 | 7.70E-34 |
| rs12662365 | 6 | 80905389 | T | -0.005 | 0.001 | 8.00E-09 |
| rs17185536 | 6 | 100620931 | C | -0.007 | 0.001 | 1.70E-14 |
| rs188968468 | 6 | 64324712 | G | 0.019 | 0.003 | 5.90E-09 |
| rs2136677 | 6 | 111176401 | C | -0.006 | 0.001 | 3.90E-11 |
| rs2208605 | 6 | 55572187 | T | -0.007 | 0.001 | 4.00E-11 |
| rs2294346 | 6 | 25689487 | G | -0.005 | 0.001 | 1.80E-09 |
| rs2299055 | 6 | 15398331 | A | 0.007 | 0.001 | 1.00E-08 |
| rs28360642 | 6 | 41667506 | A | 0.016 | 0.001 | 8.70E-48 |
| rs373498609 | 6 | 31772540 | C | 0.009 | 0.001 | 2.60E-20 |
| rs4052755 | 6 | 19914496 | C | -0.006 | 0.001 | 1.40E-11 |
| rs4472353 | 6 | 96078826 | T | -0.007 | 0.001 | 1.20E-08 |
| rs4709746 | 6 | 164133001 | C | -0.010 | 0.001 | 6.40E-17 |
| rs58263961 | 6 | 139220712 | C | -0.006 | 0.001 | 9.80E-09 |
| rs62407923 | 6 | 52391951 | C | -0.007 | 0.001 | 3.40E-09 |
| rs635243 | 6 | 139843350 | A | -0.005 | 0.001 | 1.90E-11 |
| rs6457375 | 6 | 31272612 | G | 0.006 | 0.001 | 1.30E-14 |
| rs668871 | 6 | 160769811 | C | -0.018 | 0.001 | 1.40E-111 |
| rs6941110 | 6 | 34791334 | C | -0.012 | 0.001 | 1.30E-42 |
| rs7753558 | 6 | 117523471 | C | 0.005 | 0.001 | 2.00E-11 |
| rs7756992 | 6 | 20679709 | A | 0.008 | 0.001 | 3.50E-19 |
| rs9266303 | 6 | 31329609 | G | 0.014 | 0.001 | 7.90E-37 |
| rs9272606 | 6 | 32607729 | C | 0.007 | 0.001 | 5.00E-15 |
| rs9375477 | 6 | 127204623 | A | 0.008 | 0.001 | 2.80E-12 |
| rs9379084 | 6 | 7231843 | G | 0.014 | 0.001 | 5.20E-26 |
| rs9458800 | 6 | 163753143 | T | 0.005 | 0.001 | 3.00E-09 |
| rs9480889 | 6 | 109189021 | C | 0.006 | 0.001 | 2.10E-08 |
| rs111363146 | 7 | 44801682 | T | -0.009 | 0.001 | 1.10E-15 |
| rs114949263 | 7 | 150498245 | T | -0.015 | 0.001 | 1.70E-34 |
| rs11556924 | 7 | 129663496 | C | -0.008 | 0.001 | 4.90E-18 |
| rs11764444 | 7 | 70192665 | T | -0.006 | 0.001 | 5.90E-11 |
| rs11766026 | 7 | 101907088 | C | -0.006 | 0.001 | 6.30E-13 |
| rs11770446 | 7 | 77358072 | A | -0.007 | 0.001 | 1.40E-19 |
| rs1229492 | 7 | 81564122 | T | 0.011 | 0.001 | 1.30E-32 |
| rs12701263 | 7 | 32962089 | C | -0.005 | 0.001 | 6.10E-09 |
| rs12705093 | 7 | 100477445 | A | -0.017 | 0.002 | 1.20E-31 |
| rs146296501 | 7 | 99168423 | T | -0.017 | 0.003 | 8.30E-09 |
| rs149092986 | 7 | 111624089 | T | 0.016 | 0.003 | 2.40E-08 |
| rs1534696 | 7 | 26397239 | C | -0.005 | 0.001 | 3.40E-09 |
| rs157935 | 7 | 130585553 | T | -0.012 | 0.001 | 1.40E-39 |
| rs17471520 | 7 | 27178790 | T | -0.009 | 0.001 | 9.50E-10 |
| rs2462661 | 7 | 6702311 | T | -0.007 | 0.001 | 1.00E-16 |
| rs2696887 | 7 | 135166999 | T | 0.005 | 0.001 | 1.60E-09 |
| rs2723572 | 7 | 17888587 | T | 0.005 | 0.001 | 3.20E-11 |
| rs3173833 | 7 | 150491084 | T | -0.006 | 0.001 | 4.70E-15 |
| rs38196 | 7 | 15906641 | A | -0.006 | 0.001 | 1.00E-10 |
| rs42374 | 7 | 116444070 | T | 0.006 | 0.001 | 8.60E-15 |
| rs445 | 7 | 92408370 | C | 0.012 | 0.001 | 4.80E-18 |
| rs55747707 | 7 | 73037366 | G | -0.008 | 0.001 | 3.20E-16 |
| rs62442919 | 7 | 1978384 | A | 0.008 | 0.001 | 1.50E-23 |
| rs62472728 | 7 | 143105566 | C | -0.012 | 0.002 | 7.70E-10 |
| rs6948767 | 7 | 86862750 | G | -0.014 | 0.002 | 1.50E-11 |
| rs6950023 | 7 | 97915635 | T | -0.034 | 0.001 | 1.00E-200 |
| rs71538127 | 7 | 1010801 | C | 0.009 | 0.001 | 1.50E-13 |
| rs7794048 | 7 | 46275882 | G | 0.013 | 0.002 | 4.80E-14 |
| rs7808581 | 7 | 156194185 | C | 0.005 | 0.001 | 1.00E-09 |
| rs78816009 | 7 | 74341926 | G | -0.009 | 0.001 | 1.20E-17 |
| rs849133 | 7 | 28192280 | C | -0.011 | 0.001 | 2.00E-42 |
| rs878521 | 7 | 44255643 | G | 0.006 | 0.001 | 1.10E-10 |
| rs10102041 | 8 | 59433911 | G | 0.006 | 0.001 | 5.30E-09 |
| rs10110651 | 8 | 36847115 | T | 0.007 | 0.001 | 9.50E-11 |
| rs10504255 | 8 | 59398461 | G | -0.011 | 0.001 | 3.00E-36 |
| rs11774700 | 8 | 118220270 | T | -0.006 | 0.001 | 7.30E-13 |
| rs12543287 | 8 | 42334511 | G | -0.010 | 0.001 | 2.40E-37 |
| rs12680692 | 8 | 12618225 | T | 0.006 | 0.001 | 7.30E-12 |
| rs2721195 | 8 | 145677011 | T | 0.010 | 0.001 | 4.20E-33 |
| rs4240624 | 8 | 9184231 | G | -0.022 | 0.001 | 2.30E-59 |
| rs440837 | 8 | 81461974 | A | -0.019 | 0.001 | 4.00E-80 |
| rs4739515 | 8 | 37391203 | G | -0.012 | 0.002 | 1.90E-08 |
| rs59241933 | 8 | 72521293 | C | 0.007 | 0.001 | 2.00E-12 |
| rs6557781 | 8 | 21937667 | T | -0.007 | 0.001 | 3.20E-11 |
| rs6601302 | 8 | 9239458 | T | 0.006 | 0.001 | 1.10E-11 |
| rs6999846 | 8 | 80813426 | T | -0.005 | 0.001 | 2.20E-11 |
| rs72656010 | 8 | 57122215 | T | 0.008 | 0.001 | 6.60E-12 |
| rs76767219 | 8 | 81426196 | C | -0.043 | 0.002 | 6.90E-83 |
| rs7822342 | 8 | 81246699 | A | -0.009 | 0.001 | 1.30E-30 |
| rs7828742 | 8 | 116960729 | A | 0.008 | 0.001 | 1.10E-20 |
| rs9644032 | 8 | 23414822 | T | 0.007 | 0.001 | 7.30E-18 |
| rs10114763 | 9 | 4143749 | A | -0.009 | 0.001 | 3.80E-26 |
| rs10733608 | 9 | 117148430 | G | -0.008 | 0.001 | 3.40E-24 |
| rs10811662 | 9 | 22134253 | G | -0.007 | 0.001 | 1.10E-10 |
| rs10868080 | 9 | 86626769 | T | 0.023 | 0.001 | 3.40E-125 |
| rs11515536 | 9 | 101771183 | C | -0.007 | 0.001 | 1.60E-08 |
| rs117119759 | 9 | 136212168 | G | 0.016 | 0.003 | 1.40E-09 |
| rs1397575 | 9 | 94011892 | C | 0.006 | 0.001 | 3.70E-08 |
| rs143554698 | 9 | 95538573 | C | 0.008 | 0.001 | 1.50E-12 |
| rs2416800 | 9 | 123544003 | A | 0.007 | 0.001 | 3.80E-17 |
| rs35233014 | 9 | 137268177 | C | 0.014 | 0.001 | 2.50E-46 |
| rs35234337 | 9 | 35661243 | C | 0.006 | 0.001 | 3.30E-08 |
| rs4567095 | 9 | 4309006 | C | -0.007 | 0.001 | 3.40E-15 |
| rs4917342 | 9 | 137162110 | C | -0.004 | 0.001 | 2.80E-08 |
| rs62577365 | 9 | 137019331 | C | 0.011 | 0.002 | 7.50E-10 |
| rs62580766 | 9 | 113034490 | C | -0.011 | 0.001 | 3.20E-27 |
| rs74551598 | 9 | 117177566 | A | 0.005 | 0.001 | 2.30E-09 |
| rs7847560 | 9 | 113207962 | A | -0.006 | 0.001 | 2.60E-08 |
| rs7860634 | 9 | 139089679 | G | -0.010 | 0.001 | 1.20E-30 |
| rs820503 | 9 | 6667928 | C | 0.012 | 0.001 | 4.20E-25 |
| rs874867 | 9 | 13536352 | A | -0.009 | 0.002 | 1.50E-09 |
| rs9697210 | 9 | 131468740 | G | 0.016 | 0.001 | 9.20E-45 |
| rs11002762 | 10 | 80737512 | G | 0.007 | 0.001 | 3.50E-08 |
| rs111287284 | 10 | 65273338 | A | 0.036 | 0.006 | 1.70E-10 |
| rs112924565 | 10 | 104888866 | G | -0.009 | 0.001 | 3.30E-21 |
| rs12263369 | 10 | 94823343 | C | 0.010 | 0.001 | 4.80E-36 |
| rs12770588 | 10 | 21709301 | A | -0.006 | 0.001 | 2.50E-08 |
| rs139501837 | 10 | 65352028 | C | 0.033 | 0.004 | 2.40E-19 |
| rs144642136 | 10 | 122969119 | C | 0.024 | 0.004 | 9.00E-10 |
| rs16918645 | 10 | 65346300 | A | 0.018 | 0.002 | 3.80E-27 |
| rs1772189 | 10 | 93629499 | T | 0.012 | 0.001 | 9.40E-54 |
| rs1782652 | 10 | 81074125 | T | 0.012 | 0.001 | 2.90E-50 |
| rs2915025 | 10 | 77280923 | C | -0.008 | 0.001 | 1.70E-08 |
| rs36062478 | 10 | 89722731 | T | 0.005 | 0.001 | 3.80E-08 |
| rs3737178 | 10 | 31607215 | A | 0.010 | 0.002 | 1.20E-08 |
| rs3824655 | 10 | 13370779 | G | -0.006 | 0.001 | 7.30E-14 |
| rs4746855 | 10 | 64822828 | G | -0.027 | 0.001 | 1.00E-200 |
| rs55834629 | 10 | 102441450 | A | -0.009 | 0.002 | 1.80E-08 |
| rs61853632 | 10 | 65259833 | G | -0.029 | 0.002 | 1.60E-40 |
| rs71508908 | 10 | 63911444 | C | -0.013 | 0.001 | 2.20E-20 |
| rs72783094 | 10 | 49686734 | A | 0.008 | 0.001 | 1.80E-08 |
| rs7893136 | 10 | 122926460 | C | -0.018 | 0.003 | 5.50E-10 |
| rs7903146 | 10 | 114758349 | C | 0.010 | 0.001 | 2.40E-29 |
| rs79717793 | 10 | 5262267 | G | 0.021 | 0.001 | 8.50E-84 |
| rs79829463 | 10 | 65436691 | C | -0.021 | 0.002 | 3.00E-36 |
| rs1037169 | 11 | 13361005 | T | 0.012 | 0.001 | 1.20E-41 |
| rs10769256 | 11 | 47378396 | C | 0.006 | 0.001 | 1.70E-14 |
| rs10895276 | 11 | 102083695 | C | 0.009 | 0.001 | 3.80E-25 |
| rs10902121 | 11 | 306791 | T | 0.006 | 0.001 | 2.90E-13 |
| rs1255528 | 11 | 95299962 | G | 0.007 | 0.001 | 3.60E-09 |
| rs12797706 | 11 | 65561369 | G | -0.014 | 0.001 | 3.20E-41 |
| rs12797969 | 11 | 3019216 | C | 0.006 | 0.001 | 4.20E-12 |
| rs138526953 | 11 | 118793479 | C | 0.016 | 0.003 | 1.30E-09 |
| rs174533 | 11 | 61549025 | G | 0.010 | 0.001 | 1.20E-28 |
| rs2074310 | 11 | 17421886 | T | -0.006 | 0.001 | 1.50E-12 |
| rs2292910 | 11 | 45903613 | A | 0.006 | 0.001 | 1.20E-11 |
| rs2351958 | 11 | 16248020 | C | 0.010 | 0.001 | 2.10E-38 |
| rs2924545 | 11 | 68883281 | G | 0.007 | 0.001 | 2.70E-22 |
| rs3842763 | 11 | 2179204 | G | -0.011 | 0.001 | 2.00E-28 |
| rs513249 | 11 | 114032592 | T | -0.007 | 0.001 | 7.60E-13 |
| rs55771168 | 11 | 119070949 | T | -0.007 | 0.001 | 5.70E-15 |
| rs558531 | 11 | 95086353 | G | -0.005 | 0.001 | 2.20E-09 |
| rs566217606 | 11 | 48877002 | T | -0.022 | 0.004 | 1.40E-08 |
| rs62618693 | 11 | 32956492 | C | -0.016 | 0.002 | 2.30E-16 |
| rs631695 | 11 | 69283303 | T | 0.014 | 0.001 | 6.80E-68 |
| rs10842263 | 12 | 24185398 | T | 0.009 | 0.002 | 5.90E-10 |
| rs10876451 | 12 | 53813424 | A | 0.008 | 0.001 | 4.30E-13 |
| rs11110390 | 12 | 100874901 | C | 0.009 | 0.001 | 2.10E-24 |
| rs12296154 | 12 | 21503467 | C | -0.008 | 0.001 | 8.70E-18 |
| rs12311848 | 12 | 124486851 | A | -0.010 | 0.001 | 1.10E-33 |
| rs12313762 | 12 | 57692470 | C | -0.017 | 0.001 | 2.60E-74 |
| rs145775785 | 12 | 65902265 | C | -0.023 | 0.004 | 6.10E-10 |
| rs1725788 | 12 | 131608476 | A | -0.006 | 0.001 | 3.30E-09 |
| rs2017594 | 12 | 122631208 | G | -0.007 | 0.001 | 1.40E-11 |
| rs2219828 | 12 | 21319313 | G | 0.013 | 0.002 | 9.50E-19 |
| rs2393791 | 12 | 121423956 | C | -0.015 | 0.001 | 2.00E-80 |
| rs2583920 | 12 | 66186792 | A | 0.009 | 0.001 | 2.00E-10 |
| rs374335 | 12 | 77457439 | A | 0.006 | 0.001 | 2.80E-10 |
| rs4980839 | 12 | 712980 | T | 0.005 | 0.001 | 1.10E-09 |
| rs56196860 | 12 | 2908330 | C | -0.023 | 0.002 | 4.40E-24 |
| rs5742643 | 12 | 102837863 | T | 0.009 | 0.001 | 8.70E-23 |
| rs57743625 | 12 | 21367633 | G | 0.030 | 0.001 | 1.10E-159 |
| rs60702534 | 12 | 111734681 | C | -0.008 | 0.001 | 2.80E-12 |
| rs61927768 | 12 | 50898728 | G | -0.010 | 0.001 | 4.30E-28 |
| rs7314285 | 12 | 111522026 | T | -0.036 | 0.002 | 1.50E-109 |
| rs74103241 | 12 | 77429776 | T | -0.006 | 0.001 | 2.00E-09 |
| rs75130744 | 12 | 25410741 | G | 0.027 | 0.002 | 7.30E-72 |
| rs75151534 | 12 | 20900839 | C | 0.010 | 0.001 | 4.90E-12 |
| rs76895963 | 12 | 4384844 | T | -0.073 | 0.003 | 1.20E-120 |
| rs78581485 | 12 | 101067606 | G | 0.062 | 0.004 | 5.70E-57 |
| rs7961076 | 12 | 103550623 | T | 0.009 | 0.001 | 2.60E-26 |
| rs7971398 | 12 | 46393518 | G | -0.006 | 0.001 | 7.60E-12 |
| rs7979129 | 12 | 66409705 | C | -0.006 | 0.001 | 3.00E-12 |
| rs79879123 | 12 | 93630236 | C | 0.013 | 0.002 | 2.30E-11 |
| rs112035922 | 13 | 115047464 | C | 0.008 | 0.001 | 1.90E-18 |
| rs116338429 | 13 | 114767040 | C | -0.008 | 0.001 | 2.10E-11 |
| rs12874375 | 13 | 26818902 | C | -0.006 | 0.001 | 1.70E-10 |
| rs2182784 | 13 | 51133421 | C | 0.007 | 0.001 | 1.00E-13 |
| rs41284816 | 13 | 50655989 | G | -0.031 | 0.003 | 1.50E-25 |
| rs451575 | 13 | 111301543 | C | 0.011 | 0.002 | 2.20E-09 |
| rs749170 | 13 | 22350875 | C | -0.005 | 0.001 | 7.00E-10 |
| rs9533843 | 13 | 44980150 | G | -0.005 | 0.001 | 1.80E-08 |
| rs9556404 | 13 | 95254382 | A | 0.008 | 0.001 | 2.90E-24 |
| rs112635299 | 14 | 94838142 | G | -0.092 | 0.003 | 1.00E-200 |
| rs115256445 | 14 | 100812505 | A | 0.010 | 0.001 | 1.50E-33 |
| rs11621792 | 14 | 24871926 | C | 0.019 | 0.001 | 1.80E-117 |
| rs11629105 | 14 | 23721880 | T | -0.013 | 0.001 | 5.60E-40 |
| rs12435790 | 14 | 35154381 | A | -0.010 | 0.002 | 5.80E-10 |
| rs12888084 | 14 | 89884991 | A | -0.005 | 0.001 | 1.50E-11 |
| rs13379043 | 14 | 74250126 | T | -0.010 | 0.001 | 2.60E-28 |
| rs17580 | 14 | 94847262 | T | -0.026 | 0.002 | 1.90E-42 |
| rs17780783 | 14 | 73219669 | C | 0.007 | 0.001 | 5.80E-13 |
| rs2239222 | 14 | 73011885 | A | -0.011 | 0.001 | 9.40E-35 |
| rs61758464 | 14 | 105257802 | G | -0.009 | 0.001 | 1.10E-17 |
| rs61997624 | 14 | 104582386 | C | 0.007 | 0.001 | 3.70E-10 |
| rs72683923 | 14 | 50735947 | T | -0.027 | 0.003 | 5.70E-22 |
| rs112500920 | 15 | 82507605 | T | 0.012 | 0.002 | 4.00E-11 |
| rs11634501 | 15 | 59996906 | A | -0.007 | 0.001 | 4.70E-14 |
| rs11634893 | 15 | 96644606 | G | -0.006 | 0.001 | 2.20E-13 |
| rs11856926 | 15 | 96223649 | G | 0.010 | 0.001 | 4.60E-36 |
| rs12593379 | 15 | 35180801 | G | 0.008 | 0.001 | 2.10E-19 |
| rs12594627 | 15 | 73986264 | G | -0.005 | 0.001 | 9.20E-10 |
| rs139974673 | 15 | 44027885 | T | 0.066 | 0.003 | 5.10E-147 |
| rs2218181 | 15 | 66872325 | T | 0.008 | 0.001 | 1.10E-21 |
| rs2695165 | 15 | 42073397 | C | 0.007 | 0.001 | 8.70E-18 |
| rs28510484 | 15 | 31637569 | G | 0.008 | 0.001 | 3.80E-12 |
| rs4984511 | 15 | 96704443 | C | -0.034 | 0.001 | 1.00E-200 |
| rs62011287 | 15 | 63791228 | A | 0.015 | 0.001 | 1.40E-69 |
| rs62023531 | 15 | 53168857 | C | 0.020 | 0.001 | 2.20E-47 |
| rs73401721 | 15 | 39462617 | C | -0.012 | 0.002 | 3.80E-10 |
| rs74923405 | 15 | 68312792 | C | 0.013 | 0.002 | 1.90E-10 |
| rs79391862 | 15 | 53739426 | A | 0.082 | 0.004 | 2.20E-121 |
| rs112874986 | 16 | 11884499 | G | 0.007 | 0.001 | 1.20E-15 |
| rs11642328 | 16 | 24540438 | A | 0.005 | 0.001 | 2.20E-08 |
| rs11862061 | 16 | 1858365 | T | -0.010 | 0.002 | 2.50E-12 |
| rs12928099 | 16 | 15150505 | C | -0.010 | 0.001 | 4.00E-29 |
| rs1530644 | 16 | 68396034 | G | 0.005 | 0.001 | 5.50E-09 |
| rs1985872 | 16 | 11143129 | G | 0.005 | 0.001 | 3.80E-11 |
| rs246192 | 16 | 58544295 | G | 0.007 | 0.001 | 1.40E-16 |
| rs2966084 | 16 | 81516383 | A | -0.005 | 0.001 | 1.40E-08 |
| rs34050011 | 16 | 49868513 | C | -0.006 | 0.001 | 1.20E-09 |
| rs36073017 | 16 | 87997065 | C | 0.010 | 0.001 | 1.40E-35 |
| rs3747587 | 16 | 4674954 | C | -0.010 | 0.001 | 3.00E-21 |
| rs4782568 | 16 | 83980529 | C | -0.013 | 0.001 | 4.30E-58 |
| rs4889530 | 16 | 31065918 | T | 0.008 | 0.001 | 1.60E-23 |
| rs55729432 | 16 | 88511548 | C | -0.009 | 0.001 | 4.30E-23 |
| rs7186308 | 16 | 24848471 | A | 0.006 | 0.001 | 4.60E-10 |
| rs10153315 | 17 | 79481772 | T | 0.008 | 0.001 | 2.90E-24 |
| rs11079871 | 17 | 47546542 | T | 0.006 | 0.001 | 7.40E-16 |
| rs118098353 | 17 | 7531244 | C | -0.114 | 0.003 | 1.00E-200 |
| rs12943365 | 17 | 29680526 | C | -0.009 | 0.001 | 5.50E-30 |
| rs12950511 | 17 | 47320938 | C | 0.026 | 0.001 | 1.00E-200 |
| rs139667716 | 17 | 7267699 | G | -0.047 | 0.003 | 5.50E-55 |
| rs140912942 | 17 | 7314396 | C | -0.018 | 0.004 | 4.00E-08 |
| rs141667726 | 17 | 7244137 | C | 0.259 | 0.005 | 1.00E-200 |
| rs1642792 | 17 | 7576151 | G | 0.080 | 0.004 | 9.50E-107 |
| rs17138476 | 17 | 36075605 | C | -0.007 | 0.001 | 6.60E-11 |
| rs17880847 | 17 | 7573897 | T | 0.101 | 0.004 | 3.30E-181 |
| rs1801689 | 17 | 64210580 | A | 0.038 | 0.002 | 3.00E-60 |
| rs183855978 | 17 | 7465735 | G | 0.093 | 0.003 | 1.00E-200 |
| rs183889114 | 17 | 7545273 | C | -0.025 | 0.003 | 2.30E-14 |
| rs193159515 | 17 | 7259087 | G | -0.052 | 0.002 | 1.00E-200 |
| rs2587505 | 17 | 77784268 | T | 0.006 | 0.001 | 1.30E-11 |
| rs28432811 | 17 | 47338381 | T | -0.010 | 0.001 | 1.00E-24 |
| rs3744010 | 17 | 73840243 | G | -0.010 | 0.001 | 6.50E-24 |
| rs546057177 | 17 | 7368404 | A | 0.063 | 0.004 | 1.70E-70 |
| rs55938136 | 17 | 43798360 | A | -0.005 | 0.001 | 9.10E-09 |
| rs56300212 | 17 | 65264056 | A | 0.007 | 0.001 | 7.10E-19 |
| rs56325564 | 17 | 45766771 | G | 0.015 | 0.001 | 3.20E-75 |
| rs61730843 | 17 | 7496411 | G | -0.083 | 0.006 | 2.00E-53 |
| rs72844546 | 17 | 73149850 | C | 0.010 | 0.001 | 2.80E-33 |
| rs76162631 | 17 | 7759239 | A | -0.033 | 0.004 | 2.50E-17 |
| rs79693490 | 17 | 7502608 | G | 0.122 | 0.005 | 1.70E-166 |
| rs8079418 | 17 | 17924060 | C | 0.014 | 0.001 | 3.70E-65 |
| rs9901675 | 17 | 7484812 | G | 0.068 | 0.002 | 1.00E-200 |
| rs11664106 | 18 | 2846812 | A | -0.007 | 0.001 | 7.10E-17 |
| rs12454712 | 18 | 60845884 | T | -0.010 | 0.001 | 1.00E-32 |
| rs3810027 | 18 | 60903978 | C | 0.007 | 0.001 | 1.60E-16 |
| rs4327143 | 18 | 71925113 | G | -0.008 | 0.001 | 1.30E-19 |
| rs4450488 | 18 | 54717025 | G | 0.005 | 0.001 | 5.30E-09 |
| rs7233512 | 18 | 42595076 | G | 0.007 | 0.001 | 2.00E-14 |
| rs8093364 | 18 | 55096655 | C | 0.009 | 0.001 | 2.40E-16 |
| rs111981233 | 19 | 50016479 | T | -0.021 | 0.002 | 1.60E-46 |
| rs11665716 | 19 | 33996406 | G | 0.006 | 0.001 | 2.00E-09 |
| rs11666245 | 19 | 38229926 | G | 0.017 | 0.002 | 7.00E-20 |
| rs1640269 | 19 | 2793194 | A | -0.019 | 0.001 | 9.10E-101 |
| rs202200760 | 19 | 17346854 | G | -0.073 | 0.002 | 1.00E-200 |
| rs273492 | 19 | 18237882 | G | 0.008 | 0.001 | 3.30E-19 |
| rs34255979 | 19 | 46384830 | C | -0.028 | 0.001 | 1.50E-108 |
| rs35350976 | 19 | 59023174 | A | -0.008 | 0.001 | 4.10E-16 |
| rs45512696 | 19 | 35550878 | C | -0.021 | 0.001 | 2.00E-86 |
| rs4804416 | 19 | 7223848 | T | 0.015 | 0.001 | 1.80E-75 |
| rs4805881 | 19 | 33896432 | A | -0.009 | 0.001 | 4.30E-29 |
| rs484195 | 19 | 45421877 | A | -0.005 | 0.001 | 2.30E-10 |
| rs551265709 | 19 | 17425953 | C | -0.016 | 0.002 | 4.50E-14 |
| rs60018147 | 19 | 3375572 | A | -0.014 | 0.001 | 2.60E-25 |
| rs627379 | 19 | 3745488 | C | 0.005 | 0.001 | 6.10E-09 |
| rs6510107 | 19 | 58342011 | A | -0.007 | 0.001 | 1.50E-10 |
| rs67732413 | 19 | 7191338 | G | -0.005 | 0.001 | 2.60E-09 |
| rs74183052 | 19 | 14175226 | C | 0.007 | 0.001 | 2.10E-17 |
| rs74444983 | 19 | 45745607 | T | 0.006 | 0.001 | 1.50E-08 |
| rs75748663 | 19 | 56625073 | G | -0.010 | 0.001 | 8.70E-13 |
| rs9676732 | 19 | 53833142 | G | -0.005 | 0.001 | 1.10E-12 |
| rs13042148 | 20 | 32298286 | C | 0.014 | 0.001 | 9.00E-39 |
| rs1741288 | 20 | 4102954 | G | -0.005 | 0.001 | 2.30E-11 |
| rs2618566 | 20 | 17844684 | G | 0.008 | 0.001 | 2.90E-19 |
| rs3752293 | 20 | 37570725 | C | -0.007 | 0.001 | 2.40E-16 |
| rs4810580 | 20 | 45594295 | T | 0.007 | 0.001 | 3.20E-13 |
| rs55987409 | 20 | 49569025 | C | -0.018 | 0.002 | 6.40E-28 |
| rs6029640 | 20 | 39970385 | A | -0.010 | 0.001 | 9.60E-33 |
| rs6073431 | 20 | 43040569 | C | -0.017 | 0.001 | 5.10E-92 |
| rs6090040 | 20 | 62692060 | A | -0.005 | 0.001 | 7.40E-10 |
| rs6090665 | 20 | 45993193 | T | -0.007 | 0.001 | 1.30E-09 |
| rs62217799 | 20 | 62347191 | G | 0.007 | 0.001 | 2.20E-15 |
| rs7261425 | 20 | 20068635 | C | -0.005 | 0.001 | 2.70E-08 |
| rs7271144 | 20 | 32948885 | G | -0.008 | 0.001 | 5.30E-23 |
| rs11088470 | 21 | 40689162 | A | 0.004 | 0.001 | 4.20E-08 |
| rs112078975 | 21 | 33095959 | G | 0.012 | 0.002 | 8.40E-11 |
| rs1475883 | 21 | 17591913 | G | 0.004 | 0.001 | 2.40E-08 |
| rs1129448 | 22 | 38170026 | G | -0.005 | 0.001 | 5.00E-09 |
| rs138824 | 22 | 50169852 | G | 0.009 | 0.001 | 8.00E-10 |
| rs175194 | 22 | 20163070 | G | 0.005 | 0.001 | 4.00E-08 |
| rs28412679 | 22 | 50730320 | T | -0.005 | 0.001 | 1.10E-08 |
| rs3946137 | 22 | 38988115 | A | 0.006 | 0.001 | 9.10E-12 |
| rs4820091 | 22 | 21940189 | T | -0.011 | 0.001 | 1.60E-21 |
| rs5751777 | 22 | 24267047 | C | 0.008 | 0.001 | 5.00E-20 |
| rs6005840 | 22 | 29101357 | A | 0.012 | 0.001 | 3.00E-42 |
| rs738409 | 22 | 44324727 | C | -0.023 | 0.001 | 3.10E-114 |

Abbreviations: SHBG, sex hormone binding globulin; PCOS, polycystic ovary syndrome; Chr, chromosome; SE, standard error; SNP, single nucleotide polymorphism.

**Supplementary Table 4.** Effect estimates of the associations between circulating levels of SHBG, SHBG adjusted BMI and risk of polycystic ovary syndrome in replication study.

| Traits/Methods | No. of | OR (95% CI) | P for | P for | P intercept from MR-Egger regression |
| --- | --- | --- | --- | --- | --- |
|  | SNPs |  | association | heterogeneity |  |
| SHBG |  |  |  |  |  |
| Inverse-variance weighted | 274 | 0.45 (0.30-0.68) | 1.83×10-4 | 0.003 |  |
| Simple-median | 274 | 0.31 (0.17-0.59) | 3.16×10-4 |  |  |
| Weighted-median | 274 | 0.51 (0.28-0.95) | 0.035 |  |  |
| Maximum-likelihood | 274 | 0.45 (0.30-0.68) | 1.76×10-4 |  |  |
| MR-PRESSO test | 273 | 0.45 (0.30-0.68) | 2.23×10-4 |  |  |
| MR-Egger | 274 | 0.76 (0.36-1.60) | 0.469 |  | 0.101 |
| SHBG adjusted BMI |  |  |  |  |  |
| Inverse-variance weighted | 418 | 0.50 (0.33-0.75) | 8.87×10-4 | 0.008 |  |
| Simple-median | 418 | 0.35 (0.17-0.69) | 0.002 |  |  |
| Weighted-median | 418 | 0.87 (0.46-1.63) | 0.66 |  |  |
| Maximum-likelihood | 418 | 0.50 (0.33-0.75) | 9.39×10-4 |  |  |
| MR-PRESSO test | 418 | 0.50 (0.33-0.75) | 9.66×10-4 |  |  |
| MR-Egger | 417 | 0.97 (0.50-1.91) | 0.936 |  | 0.015 |

Abbreviations: CI, confidence interval; No, Number ; MR, Mendelian randomization; MR-PRESSO test, MR-Pleiotropy RESidual Sum and Outlier test; OR, odds ratio; SHBG, sex hormone binding globulin; PCOS, polycystic ovary syndrome; SNP, single nucleotide polymorphis
